# Nitrous oxide as a function of oxygen and archaeal gene abundance in the North Pacific

**DOI:** 10.1038/ncomms13451

**Published:** 2016-12-01

**Authors:** Mark Trimmer, Panagiota-Myrsini Chronopoulou, Susanna T. Maanoja, Robert C. Upstill-Goddard, Vassilis Kitidis, Kevin J. Purdy

**Affiliations:** 1 School of Biological and Chemical Sciences, Queen Mary University of London, London E1 4NS, UK; 2 School of Marine Science and Technology, Ridley Building, University of Newcastle, Newcastle upon, Tyne NE1 7RU, UK; 3 Plymouth Marine Laboratory, Prospect Place, West Hoe, Plymouth PL1 3DH, UK; 4 School of Life Sciences, University of Warwick, Coventry CV4 7AL, UK

## Abstract

Oceanic oxygen minimum zones are strong sources of the potent greenhouse gas N_2_O but its microbial source is unclear. We characterized an exponential response in N_2_O production to decreasing oxygen between 1 and 30 μmol O_2_ l^−1^ within and below the oxycline using ^15^NO_2_^−^, a relationship that held along a 550 km offshore transect in the North Pacific. Differences in the overall magnitude of N_2_O production were accounted for by archaeal functional gene abundance. A one-dimensional (1D) model, parameterized with our experimentally derived exponential terms, accurately reproduces N_2_O profiles in the top 350 m of water column and, together with a strong ^45^N_2_O signature indicated neither canonical nor nitrifier–denitrification production while statistical modelling supported production by archaea, possibly via hybrid N_2_O formation. Further, with just archaeal N_2_O production, we could balance high-resolution estimates of sea-to-air N_2_O exchange. Hence, a significant source of N_2_O, previously described as leakage from bacterial ammonium oxidation, is better described by low-oxygen archaeal production at the oxygen minimum zone's margins.

## Abstract (web summary)

Understanding the production processes behind oceanic sources of nitrous oxide (N_2_O), a potent greenhouse gas, is of critical importance. Here, the authors reveal an archaeal-mediated N_2_O production pathway in the North Pacific, which increases exponentially with decreasing oxygen.

## Graphical abstract

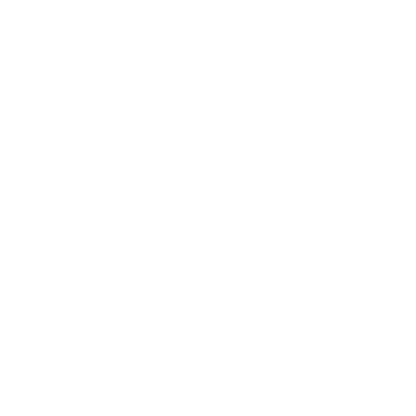

Permanent oceanic oxygen minimum zones (OMZs) are significant sources of tropospheric N_2_O (∼0.8–1.35 Tg N yr^−1^ or 20–75% of oceanic total^1^ excluding coasts^2^), a potent greenhouse gas that also plays key roles in atmospheric chemistry^1^,^3^. Oversaturation of N_2_O within an OMZ is undoubtedly due to microbial activity but the precise nature of the organisms and biochemistry responsible for its production remain to be fully characterized. Some N_2_O production in OMZs has been ascribed to classic, canonical denitrification at the base of an oxycline^4^,^5^, whereas deeper into the functionally anoxic core of an OMZ, there is also net reduction of N_2_O to N_2_ by denitrification^5^,^6^,^7^. Above the anoxic core, production of N_2_O is traditionally described as a single function of bacterial nitrification under oxygen stress, with the yield of N_2_O, from the oxidation of ammonium, increasing as oxygen declines^8^,^9^,^10^. More recently, there have also been suggestions for a coupling (both inter- and intracellular) between denitrification and nitrification as a means of N_2_O production and there is growing evidence for a direct contribution from the Archaea to this process^2^,^4^,^11^,^12^,^13^.

The documented thickening of OMZs across the world^14^ has not only increased the volume of low oxygen waters with a potential to produce N_2_O but such thickening also makes that N_2_O more readily exchangeable with the atmosphere. There is clearly therefore a need to improve our understanding of the production of this atmospherically potent N_2_O at both the margins of OMZs and beyond in hypoxic, coastal waters^2^. Many have taken the linear negative correlation often observed between N_2_O (oversaturation relative to atmospheric equilibration) and O_2_ in surface waters to indicate bacterial nitrification as the predominant source of N_2_O (that is, the N_2_O anomaly versus apparent oxygen utilization and see ref. 15). Classic bacterial nitrification as the source of N_2_O was corroborated by early observations with a pure culture of the ammonia-oxidizing bacterium *Nitrosomonas* sp., where the yield of N_2_O per mole of ammonium oxidized increased exponentially as oxygen declined^8^. This regulatory effect of O_2_ on N_2_O production in the ocean is now widely accepted (some 100 papers citing^8^ in relation to ocean N_2_O production), though its exponential form has not, to the best of our knowledge, been characterized experimentally in the ocean below 30 μmol O_2_ l^−1^ (ref. 13). In addition, there are few, if any, ocean-based experimental data to substantiate this single physiological response. For example, incubation of OMZ oxycline waters with ^15^NH_4_^+^ might be expected to yield predominantly ^46^N_2_O, that is, both N in N_2_O derived from NH_4_^+^, (^15^NH_4_^+^→^15^NH_2_OH→^15^NO+^15^N_2_O→^15^NO_2_^−^), if classic oxygen-stressed, bacterial-nitrifier N_2_O production was active, but this is not the case^4^. Rather, this pathway of bacterial-nitrifier N_2_O production has routinely been used for the purposes of mass balance or to simply rationalize water column distributions of N_2_O without any supporting experimental evidence^9^,^10^.

In addition to the poorly substantiated mechanistic basis for bacterial-nitrifier N_2_O production in the ocean, it is now evident that the Archaea are widespread in the ocean, playing significant roles in key processes such as nitrification and potentially the production of N_2_O (refs 12, 13, 16, 17). The Thaumarchaeota, the archaeal phylum that encompasses the ammonium oxidizing archaea (AOA^18^), are commonly found in low-oxygen waters at the margins of an OMZ. AOA abundance decreases as oxygen concentrations rise towards air saturation in the upper mixed layers of the ocean^13^,^19^ and have been shown to also decrease as oxygen practically disappears at the oxic to anoxic interface at the core of an OMZ^6^. Thus, lower-oxygen waters appear to be an important niche for at least some Thaumarchaeota groups^20^. It is well established, at least for laboratory cultures, that bacterial ammonia oxidizers can produce N_2_O directly: either as a by-product of nitrification^8^ or through nitrifier–denitrification^21^. It is now evident that some AOA can also produce N_2_O during nitrification, but probably not via a nitrifier–denitrification-like process. New models of archaeal ammonia oxidation indicate a key role for NO produced by nitrite reductase encoded by the gene *AnirK*^22^ providing a clear link to the production of N_2_O, possibly via hybrid N_2_O formation^22^,^23^,^24^,^25^. A role for AOA-mediated N_2_O production has been suggested in the oceans, with process, natural abundance isotope values and molecular data supporting this idea^12^,^20^,^26^,^27^. There has, however, been no formal experimental characterization of N_2_O production at oxygen concentrations representative of the margins of an OMZ (<62.5 μmol O_2_ l^−1^) where N_2_O accumulates^2^,^28^ and/or the abundance of AOA (or any other candidate organisms) in representative samples of the ocean^13^.

Here we provide experimental evidence, with samples from the eastern tropical North Pacific (ETNP), which clearly links an exponential increase in N_2_O production to decreasing oxygen between 1 to 30 μmol l^−1^ and to archaeal gene abundance, together with a ^15^N pattern in the N_2_O that is reconciled by an archaeal mode of production.

## Results

### Water column characteristics

**Figure 1:**
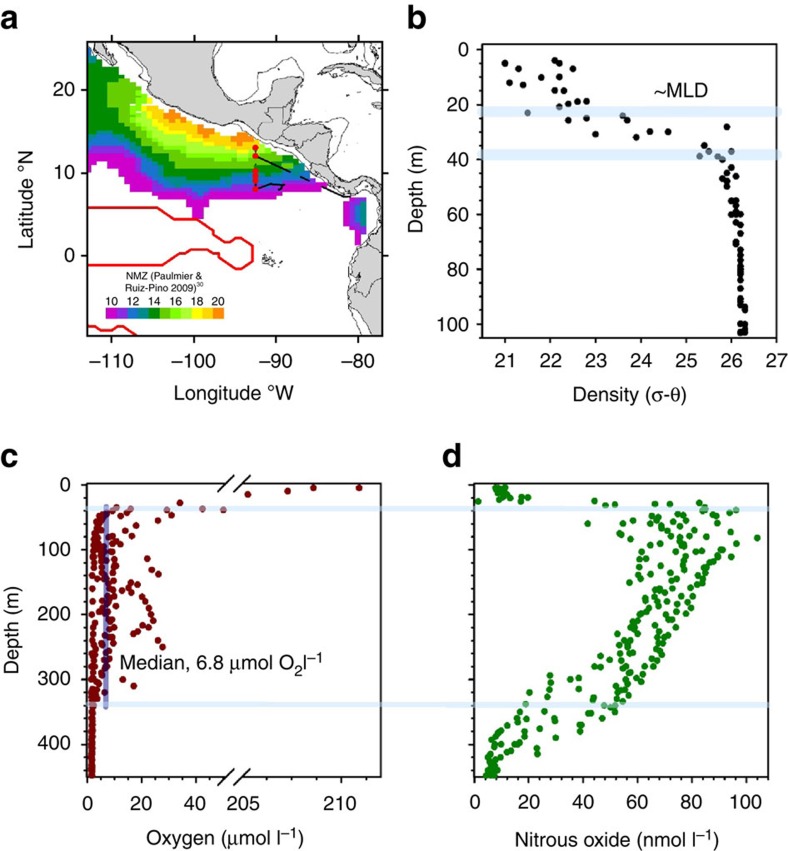
Open ocean OMZ sites and main water column profiles. (**a**) Offshore transect (bold black line with sites as filled red circles) through the nitrite maximum zone (NMZ) from 13 °N, at approximately 120 km off the coast, to 8 °N, at 670 km offshore. The red line marks the boundary of the permanent OMZ (min. O_2_<20 μmol l^−1^,) and the colour indicates the intensity of the nitrate deficit (>10 μmol l^−1^) within the NMZ (reprinted from ref. 80). (**b**) Density as a function of depth: upper light-blue line, approximate mixed layer depth (MLD); and lower light-blue line, base of the pycnocline. (**c**) Oxygen dropped rapidly in the pycnocline to ≤50 μmol O_2_ l^−1^ at its base (upper light-blue line) but remained above the limit of detection for the Seabird Sensor (∼ 1.6 μmol l^−1^) to 340 m (lower light-blue line). Within these boundaries (light-blue lines in **c**,**d**), oxygen was present at 6.8 μmol l^−1^, on average (median, vertical dark-blue), and we measured a broad peak in N_2_O (**d**). Profiles to 700 m are given in Supplementary Fig. 1. Panels **b**–**d** were drawn in SigmaPlot (Systat Software, San Jose, CA, USA).

Along our offshore transect through the nitrite maximum zone (NMZ, Fig. 1a and Supplementary Table 1), the mixed layer depth (MLD) extended down to approximately 20 to 25 m and then the density increased steadily to a sharp inflection at 35 to 40 m, marking the base of the pycnocline (Fig. 1b). Oxygen dropped rapidly in the pycnocline to ≤50 μmol O_2_ l^−1^ at its base but remained above the limit of detection for the Seabird Sensor to approximately 340 m (Fig. 1c). Within these low-oxygen waters (6.8 μmol O_2_ l^−1^, median value) we measured a broad, characteristic peak in N_2_O of up to 104 nmol N_2_O l^−1^ (Fig. 1d). Deeper, at around 350 m, oxygen became comparatively constant, with the functionally anoxic core of the OMZ^7^, where both the secondary nitrite maxima and N_2_O minima were measured, occurring deeper still at 400 to 450 m (Supplementary Fig. 1a). We found that the waters at all of the depths described so far were supersaturated with CO_2_ (Supplementary Fig. 1b), with CO_2_ being strongly correlated with N_2_O over the top 45 m (Supplementary Fig. 1c).

We set our observations for N_2_O into the wider context of the eastern tropical North Pacific by comparing them with profiles in the MEMENTO database^29^ (Supplementary Fig. 2). Although there is considerable variation in the profiles, peak concentrations of 60 to 100 nmol N_2_O l^−1^ at 100 m are present between approximately 0 °N to 22 °N and out to approximately 155 °W, with this triangle roughly marking the extent of the nitrite maximum zone (NMZ), within the wider boundary of the OMZ as a whole^30^.

### Nitrous oxide production as a function of oxygen

**Figure 2:**
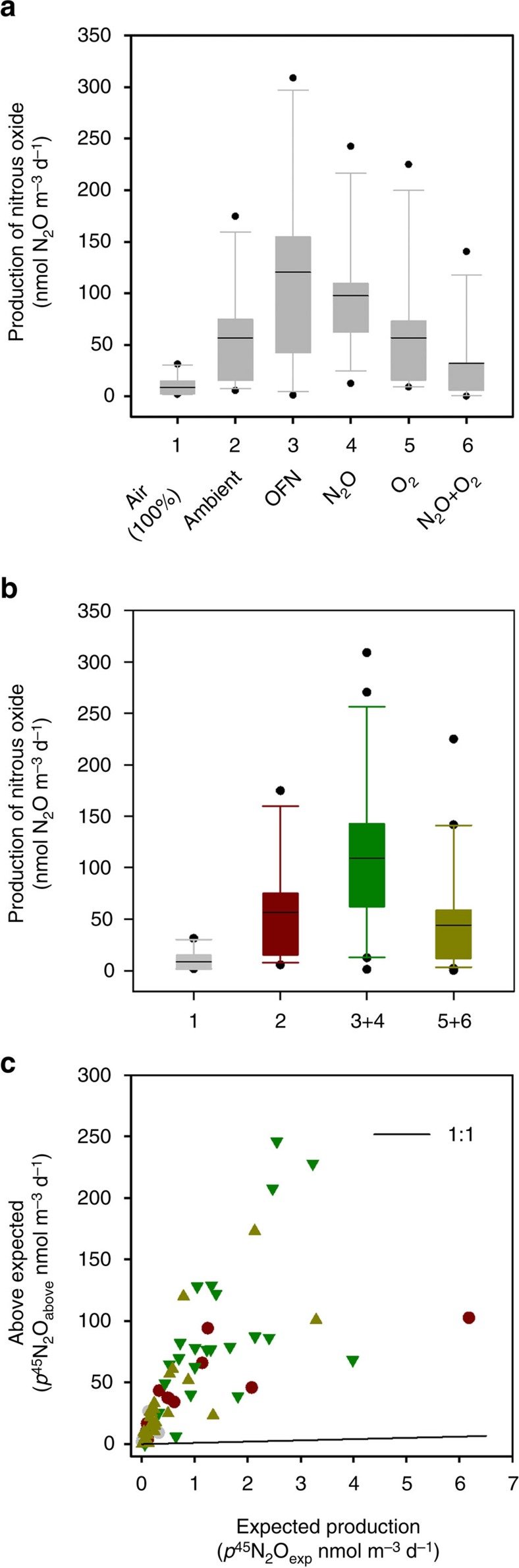
Total production of N_2_O from equations (1, 2, 3, 4, 5) and its ^15^N-labelling. (**a**) All oxygen treatments, including any inhibitory effect of N_2_O (50 nmol N_2_O l^−1^ crossed with O_2_). (**b**) As this latter treatment had no effect, the data were pooled by their comparative oxygen concentrations (3+4 and 5+6; see Table 1). Each box in **a** and **b** shows the 25th and 75th percentile, overall spread in the data and median value (horizontal line). In both **a** and **b**, the effect of treatment is highly significant (*P*<0.0001). (**c**) Production of *p*^45^N_2_O is clearly above that expected (*p*^45^N_2_O_exp_) from denitrification of NO_2_^−^ (see the ‘Methods' section) in each treatment and the symbol colours in **c** are the same as in **b** (grey=100% air; red=ambient; green=OFN and N_2_O; olive-green=O_2_ and N_2_O+O_2_). Drawn in SigmaPlot (Systat Software, San Jose, CA, USA).

We measured the production of ^15^N_2_O in incubations with ^15^NO_2_^−^ at two depths at each of our six offshore sites (*n*=12 groups of experiments). To generate natural variation in ambient water column oxygen and nitrous oxide concentrations, each depth was either within or beneath the oxycline (Supplementary Table 2). Each group of experiments comprised up to six oxygen treatments, giving us 70 independent observations (*n*=70) for the production of N_2_O as a function of oxygen (Table 1). Production of N_2_O (*p*N_2_O_total_ equations 1, 2, 3, 4, 5) was strongly modulated by the level of oxygen in each treatment (likelihood ratio test for treatment, degree of freedom 5, *χ*^2^ 38.365, *P*<0.0001 (ref. 7) and degree of freedom 3, *χ*^2^ 34.688, *P*<0.0001 for the full and pooled data sets, respectively) and was maximal in waters degassed with nitrogen (Fig. 2a,b). In addition, the ^15^N_2_O produced in each experiment was predominantly single labelled ^45^N_2_O (that is, only having one ^15^N), at a level far above (81%, on average) that expected for denitrification (4–23%; Supplementary Table 1) given the ^15^N labelling of the NO_2_^−^ pool (Fig. 2c, equations 2 and 3 in the ‘Methods' section and Supplementary Table 1). Given the labelling of produced N_2_O, we could only ascribe 19% of the N_2_O to the reduction of exogenous nitrite (N_2_O_exogenous_, equation 1) with the large majority (81%) of the N_2_O being due to some form of endogenous coupling (N_2_O_endogenous_, equation 4 and see below).

**Figure 3:**
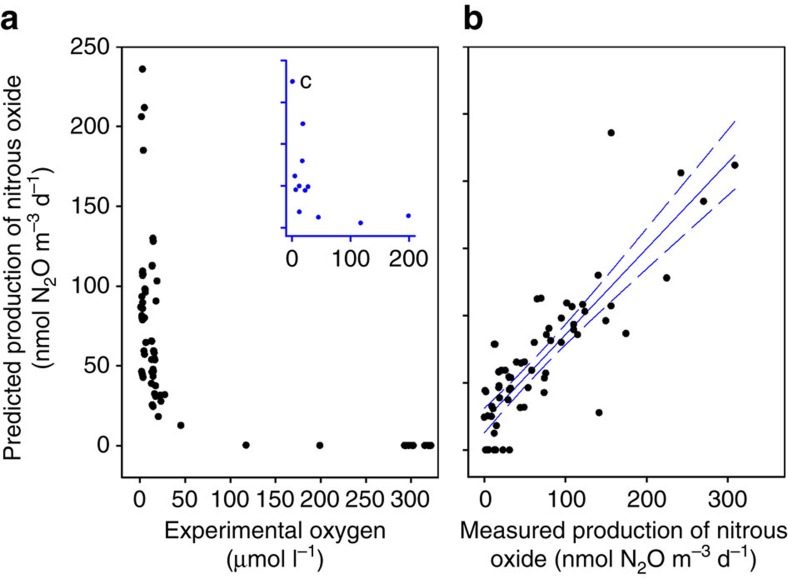
Production of total N_2_O from equations (1, 2, 3, 4, 5) as an exponential function of decreasing O_2_. Output from the nonlinear mixed-effects model M2 (Table 2) with (**a**) predicted production of N_2_O as a function of measured oxygen in each incubation bottle and (**b**) the same predicted production of N_2_O as in **a**, repeated as a function of measured production (95% confidence interval). Inset (**c**) the original data for the 12 ambient, unadulterated incubations (same units as **a**). Overall, by allowing any natural variation in the production of N_2_O (intercept *a*) to vary randomly between the 12 experiments, the nonlinear mixed-effects model captures the exponential increase in N_2_O production below 30 μM oxygen well. See main text for further explanation and Supplementary Fig. 3 for the complete model output with individual fits for each experiment and overall population parameter estimates. The latter of which we then use as input to a 1D model of water column N_2_O production. Drawn in SigmaPlot (Systat Software, San Jose, CA, USA).

Ambient controls were used to represent N_2_O production in unadulterated water, that is, straight from the sampling bottles on the conductivity-temperature-depth rosette and, in these, oxygen concentrations ranged naturally from 1 to 199 μmol O_2_ l^−1^. In addition, the concentration of oxygen set at each level of the treatment varied (coefficient of variation (CV) of 4% to 35% for all the levels) across the 12 groups of experiments (Table 1). To account for this oxygen gradient in the 12 groups of experiments, and any natural variation in the water samples, we used a nonlinear mixed-effects approach to model the production of total N_2_O as an exponential function of decreasing oxygen (Table 2 and Supplementary Fig. 3). The most parsimonious model (M2) required only a random intercept (*a*), which allowed the overall magnitude of total N_2_O production to vary randomly between the 12 groups of experiments, while keeping the response to oxygen (*b*) constant. The mixed-effects model captures the data well (Fig. 3). The overall exponential increase in production of N_2_O with oxygen decreasing below 30 μmol O_2_ l^−1^ is not only consistent with N_2_O accumulating below 30 μmol O_2_ l^−1^ in the water column (Fig. 1d) but also with distributions seen in many parts of the tropical North Pacific (as above, Supplementary Fig. 2). We also measured the production of N_2_O over time at two oxygen concentrations (Supplementary Fig. 4) to check whether our 72 h incubation overestimated production. Where production was strongest (30 and 56 nmol m^−3^ d^−1^) and representative of the 12 main experiments (median 58 nmol m^−3^ d^−1^, <30 μmol O_2_ l^−1^), it was approximately linear over the first 18 h and then decreased over time. If anything, our single time point incubations may have underestimated N_2_O production slightly. Overall, however, we conclude that our experiment captured the regulation of N_2_O production by oxygen in the ocean. None of the incubations produced any ^15^N labelled N_2_, not even at 1 μmol O_2_ l^−1^.

Nitrification was also clearly active in the water column. We measured a primary nitrite maximum (Supplementary Fig. 1a) and, in ambient samples of water, with oxygen at 1 to 23 μmol O_2_ l^−1^, significant oxidation of both ammonium and nitrite (2.4 nmol N l^−1^ d^−1^ and 19.1 nmol l^−1^ d^−1^ on average for each, respectively), and net nitrification of up to 8.2 nmol N l^−1^ d^−1^ (Supplementary Table 2).

### Variation in nitrous oxide production with gene abundance

By allowing the magnitude of total N_2_O production (*a*) to vary randomly between the 12 groups of experiments the mixed-effects model was able to derive an overall ‘population' estimate for the response to decreasing oxygen. The magnitude of any deviation from this estimate, that is, the random intercept, can be used to further explore relationships with other explanatory variables that could potentially account for that random variation in the magnitude of N_2_O production. For example, there was a clear effect of ambient oxygen concentration in the water column: with samples collected from above 30 μmol O_2_ l^−1^ producing less N_2_O, on average, to those collected from below 30 μmol O_2_ l^−1^ (Supplementary Fig. 5a). In agreement with a growing number of cases in the literature, we were not able to detect either of the bacterial ammonia mono-oxygenase genes, β-*amoA* or γ-*amoA*, but we did find high abundance of archaeal *amoA* (*AamoA*, 5.6 × 10^3^ copies ml^−1^, on average). We also quantified archaeal *nirK* (*AnirK*, 2.2 × 10^3^ copies ml^−1^, on average) and bacterial *nirK* and *nirS* (13 × 10^3^ and 0.7 × 10^3^ copies ml^−1^, on average, respectively), genes coding for the potential to reduce NO_2_^−^ (Supplementary Fig. 6). Both pairs of either archaeal or bacterial functional genes were positively correlated with each other but the pairs were ordinated separately in the samples collected (Supplementary Fig. 7). There was no visual indication of a pattern in the deviation of the random intercept and abundance of either bacterial *nirK* or *nirS* (Supplementary Fig. 5b) but there was a positive pattern in the abundance of *AnirK* and *AamoA* (Supplementary Fig. 5c) that we explore further.

**Figure 4:**
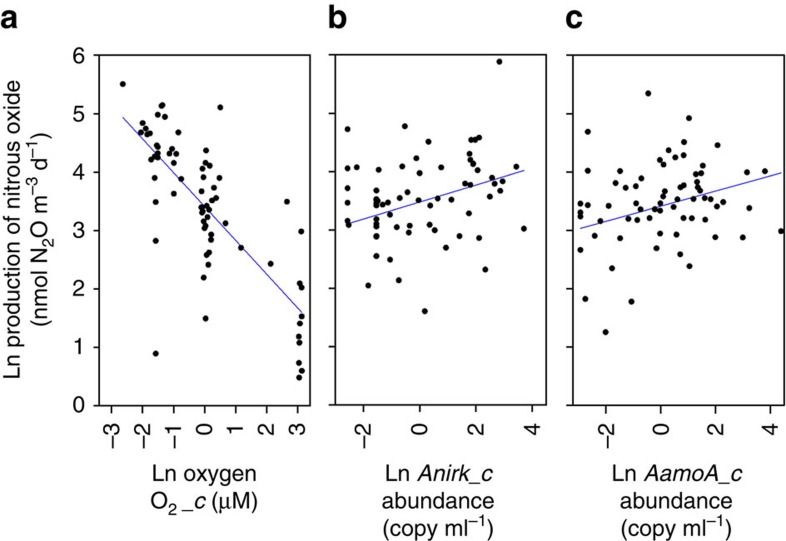
Multiple linear regression for the production of N_2_O from equations (1, 2, 3, 4, 5) as a function of O_2_ and archaeal functional gene abundance. (**a**) N_2_O production increasing with decreasing experimental oxygen (from Fig. 3a) and (**b**,**c**) production of N_2_O increasing as a function of the abundance of both *AnirK* and *AamoA*, that is, archaeal functional gene abundance accounts for some of the random variation in N_2_O production in the original nlme analysis (Table 2, Supplementary Fig. 5). The model (M14, Supplementary Table 3) suggested equal influence on the data from *AnirK* and *AamoA*, which the overwhelming ^14^N and ^15^N labelling of the N_2_O produced corroborated further. In **a**–**c**, the *x* axis data have been linearized and centred, see the ‘Methods' section. Drawn in SigmaPlot (Systat Software, San Jose, CA, USA).

The fully parameterized (oxygen combined with all four candidate genes) nonlinear mixed-effects models failed to converge and to explore the production of N_2_O as a function of both oxygen and functional gene abundance further, we log-transformed the data and proceeded with multiple linear regression (Supplementary Table 3). Oxygen alone had a highly significant negative effect on the production of N_2_O (Fig. 4a, Supplementary Table 3: M7 versus M6). Despite the comparatively similar abundance of bacterial *nirS* to the archaeal genes and the greater gene abundances for *nirK*, neither *nirS* nor *nirK* improved the fit of the model over oxygen alone, either singularly or when combined (Supplementary Table 3: M7 versus M8, M9, M10). Only inclusion of *AamoA* and/or *Anirk* in the model indicated any significant influence on the overall production of N_2_O (Supplementary Table 3: M7 versus M12, M13, M14, M15). As the model could not distinguish between the influence of either *AamoA* or *Anirk* on the distribution of the data, we would conclude that the most parsimonious explanation of our data is maximal production of N_2_O at lowest oxygen, combined with a positive influence from the abundance of both archaeal functional genes (Fig. 4). To confirm that the rate of N_2_O production was reasonable for the abundance of genes *AnirK* and *AamoA* detected, we calculated a per copy rate (equivalent to a per cell rate) for median N_2_O production (58 nmol m^−3^ d^−1^), below 30 μmol O_2_ l^−1^. Accordingly, 2 and 5 attomol N_2_O per copy per hour for the two genes, respectively, is representative of published rates (2–58 attomol N_2_O per cell per hour (ref. 31)).

### Depth-integrated N_2_O production and sea to air exchange

**Figure 5:**
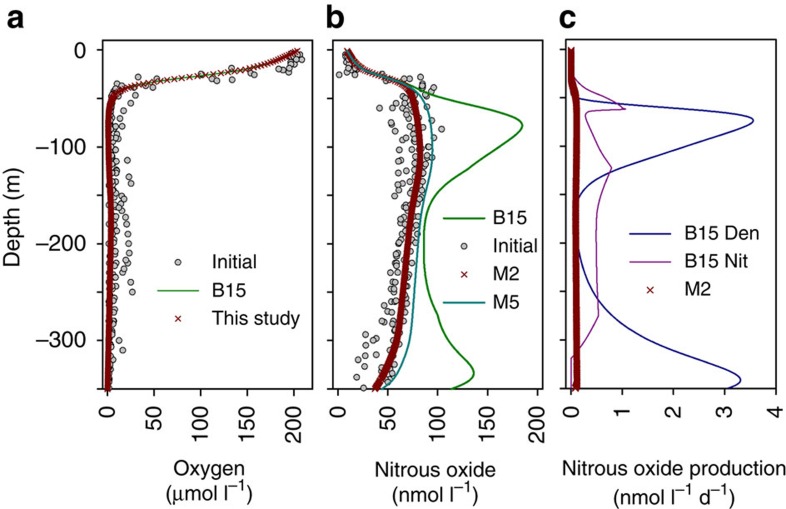
Comparison of our model output after 30 days with that of the Babbin model. (**a**) Concentration profiles for O_2_ and (**b**) for N_2_O. In **b**, N_2_O is either produced according to B15 from Babbin *et al.*^5^ or according to two parameterizations of equation (7), using either M2 or M5 (Table 2). In M2, we ascribe the majority of N_2_O production (*p*N_2_O_total_) to a 1:1 coupling, whereas, with M5, we assume that all of the N_2_O comes from a random mixing of ^14^N and ^15^N labelled NO (*p*N_2_O_total_′); note the better fit to the data with M2. (**c**) N_2_O production sources according to either a single response to oxygen, equation (7), parameterized with M2, or according to B15; where the two sources of nitrification and denitrification are B15 Nit and B15 Den, respectively. Initial conditions in both models were set using the mean profile from all of our observations but we show the output against all the data here to illustrate the goodness of fit of our simpler model. Drawn in SigmaPlot (Systat Software, San Jose, CA, USA).

We used the coefficients from our nonlinear mixed-effects models (M2 and M5, Table 2) as input to a simple one-dimensional (1D) model of N_2_O, coded in R (ref. 32, see the ‘Methods' section). The objective of this was to test whether a single O_2_-dependent, N_2_O production process could sustain the observed N_2_O maximum at∼100 m depth. Over a 30 day run (1 min time-step), parameterization with M2 maintained the initial steady state conditions (Fig. 5) without any marked accumulation (+0.04% d^−1^) of N_2_O, whereas, with M5, we saw significant N_2_O accumulation. Note that with M2 the vast majority of the N_2_O is assumed to come from a 1:1 coupling (equations 1, 2, 3, 4, 5), whereas with M5 we assume random mixing of ^14^NO and ^15^NO upstream of N_2_O production (equations 6 and 7). Given the better fit between our 1D model parameterized with M2, and our measured profiles of water column N_2_O, we favour and proceed with M2 (see the ‘Discussion' section). The sea-surface N_2_O concentration in our model was fixed (Dirichlet boundary conditions) based on the average concentration from our observations (9.3 nmol N_2_O l^−1^). Sea-air exchange (efflux to the atmosphere) was therefore implicit in our model and this efflux of N_2_O was sustained by a positive concentration gradient with depth, implying an upward flux of N_2_O. Since the model water column was losing N_2_O at the surface, it is implicit that this upward flux of N_2_O should compensate for the gas exchange loss term (that is, equal sea-air exchange). Therefore, we calculated the flux over the upper 2 and 5 m of the model water column to derive the model sea-air flux. These depths were chosen to represent the turbulent layer near the sea surface given the relatively low average wind speed (5 m s^−1^, Supplementary Fig. 8b). The resulting sea-air flux of N_2_O was 17.9 μmol N_2_O m^−2^ d^−1^ and 16.0 μmol N_2_O m^−2^ d^−1^ over 2 and 5 m, respectively. We compare this with an estimate from our high-resolution *p*CO_2_ data (*n*=4,820) and water column profile data (‘Methods' section and Supplementary Figs 1 and 8). Accordingly, our average estimate for CO_2_ exchange was 7.2 mmol CO_2_ m^−2^ d^−1^ (95% confidence interval of 6.9 to 7.4), with an equivalent exchange for N_2_O of 17 μmol N_2_O m^−2^ d^−1^ (95% confidence interval of 15.6 to 17.5); the latter agreeing well with the 1D model estimate for sea to air exchange of 16.0–17.9 μmol N_2_O m^−2^ d^−1^. Our experimental manipulation of oxygen, combined with a mixed-effects modelling approach has enabled us to parameterize a simple, single process 1D model that reproduces the pattern of N_2_O observed in the top 350 m of the tropical North Pacific.

## Discussion

Here we have shown experimentally that production of N_2_O increases exponentially below 30 μmol O_2_ l^−1^ and that this sensitivity to oxygen holds along a 550 km offshore transect through the OMZ of the eastern tropical North Pacific. Further, variation in the overall magnitude of N_2_O production correlates positively with the abundance of archaeal functional genes that potentially play a role in that production of N_2_O. Parameterizing a simple 1D model with our experimentally derived exponential terms enabled us to model accurately the distribution of N_2_O over the top 350 m of the water column and, with a single response to oxygen, we could balance our estimates of sea to air exchange for N_2_O.

Here we were following up on our previous work in the Arabian Sea^4^ where the vast majority of N_2_O production could, apparently, be accounted for by canonical denitrification of ^15^N-NO_2_^−^. Accordingly, we enriched the NO_2_^−^ pool with ^15^N in excess of 87 atom% and if denitrification were the dominant source of N_2_O, and the labelling of N_2_O random and binomially distributed (Supplementary Table 1), then we would have expected a maximum of 23% of the resulting N_2_O to be single-labelled, ^45^N_2_O. In contrast, however, we measured far more ^45^N_2_O than expected throughout (Fig. 2c). Put simply, the majority of N in the N_2_O produced was actually ^14^N that was not derived from our ^15^N-NO_2_^−^ tracer. Although nitrite is known to have a stimulatory effect on the expression of *nirK*, at least in pure cultures of *Nitrosomonas europaea*, this is at nitrite concentrations of ∼10 mM, far higher than that applied here (10 μM), which is typical of ^15^N tracer work for ^15^N-gas production^6^,^33^,^34^.

One possibility is that dilution of the ^15^N-NO_2_^−^ pool occurred via oxidation of ^14^N-NH_4_^+^ to ^14^N-NO_2_^−^ and ^15^N-NO_2_^−^ to ^15^N-NO_3_^−^, as this would increase the chance of any subsequent denitrification producing ^45^N_2_O, relative to ^46^N_2_O. For this to be plausible, however, ^14^N-NO_2_^−^ production would need to make ∼20 μmol l^−1^ at the start of the incubation. We did measure significant nitrification activity (Supplementary Table 2) similar to that in the south eastern Pacific^35^. Such activity, however, could only turn over approximately 0.2% d^−1^ of the NO_2_^−^ during our incubations, which would have had a negligible effect on the ratio of ^45^N_2_O to ^46^N_2_O produced. Alternatively, there could be a direct coupling between externally applied ^15^NO_2_^−^ and internally supplied ^14^NO_2_^−^, or other ^14^N intermediate (for example, ^14^NO), from ammonia oxidation^22^,^23^, as shown for bacterial nitrifier–denitrification^31^. Such a coupling has been argued as a possible route for N_2_O production in the oligotrophic North Pacific^36^. Here we were not able to detect the bacterial ammonia mono-oxygenase genes, β-*amoA* or γ-*amoA*. Given this apparent absence of any bacterial, nitrifier–denitrifier genomic potential, along with the overestimation of N_2_O production in our model (M5, Fig. 5b), through such a path, we would refute bacterial nitrifier–denitrification in this setting. Finally, oxidation of NO_2_^−^ has been measured simultaneously with NO_3_^−^ reduction at up to ∼16 μmol O_2_ l^−1^ (ref. 35) with comparable activity of 14 nmol N l^−1^ d^−1^ and 21 nmol N l^−1^ d^−1^ for NO_2_^−^ and NO_3_^−^, respectively (median values). Although we did not quantify NO_3_^−^ reduction, if its activity were comparable to our measured rates of NO_2_^−^ oxidation it would have the same negligible effect on the ^15^N labelling of the NO_2_^−^ pool.

Our oxygen experiments, combined with statistical modelling, indicate that highest N_2_O production is best explained by low oxygen together with a high abundance of both *AnirK* and *AamoA*. Hence, the patterns in the genomic potential for both the reduction of NO_2_^−^ (our source of ^15^N) and oxidation of ammonium (as a source of ^14^N, possibly NH_2_OH which has been reported as integral to archaeal ammonia oxidation^22^,^23^), combined with the concentration of O_2_, account for the predominant production of ^14^N and ^15^N labelled ^45^N_2_O. We could find no significant relationship for bacterial *nirK* and *nirS*. Admittedly, gene abundance (and by extrapolation cell number) does not necessarily confer a direct role on that gene for a measured process. Yet, similar positive relationships between Thaumarchaeota cell abundances and nitrification potentials are present in the low-oxygen waters of the Baltic, which, along with exponential increases in *AamoA* abundance below 100 μmol O_2_ l^−1^ in the Atlantic, suggest maintenance of active populations of Thaumarchaeota in low-oxygen waters^13^,^19^.

Overall, our data agree with the growing body of evidence for archaeal-mediated N_2_O production^12^,^13^. Although the precise biochemistry of this pathway is unknown, recent reports showing a key role for NH_2_OH and NO in Thaumarchaeotal ammonia oxidation provide support for a 1:1 coupling in N_2_O production in these organisms^22^,^23^ in support of our favoured model (Fig. 5b, M2, equations 1, 2, 3, 4, 5). As the nitrifying Archaea are better adapted to low oxygen compared with their bacterial analogues, it is unlikely N_2_O production occurs via an ammonia oxidizing bacterial type biochemical leak^37^. Rather, the metabolism of the potential precursor substrates (NH_2_OH and NO) in hybrid N_2_O formation might represent a genuine route of energy conservation^22^. Given the apparent absence of *Nor* or its equivalent in the archaea, we have to assume that our exogenous N_2_O (19% of total N_2_O production, on average) was produced through canonical denitrification operating as far as N_2_O but it remains to be proven whether the ability to metabolize NO further to N_2_O is truly absent from archaea in the ocean. Furthermore, modelling of N_2_O production in the ocean^15^ suggests that the oxycline is important, but has linked this N_2_O production primarily to bacterial ammonium oxidation (nitrification). Here we show that this is not the case and a major driver of N_2_O production in the ocean is likely to be archaeal hybrid N_2_O formation.

Although in principle, the regulatory effect of oxygen on N_2_O production in the ocean is widely accepted (some 100 papers citing Goreau *et al.*^8^ in relation to ocean N_2_O production), its exponential form has not, to the best of our knowledge, been characterized experimentally in the ocean below 30 μmol O_2_ l^−1^ (ref. 13). The basic linear ΔN_2_O/AOU relationship is frequently used to model the distribution of N_2_O across the oceans, where the slope represents the yield of N_2_O per mole of O_2_ consumed (see ref. 15 for a full discussion). This theoretical yield varies widely (0.076 to 0.31 nmol N_2_O μmol^−1^ O_2_ consumed) and can struggle to capture the full dynamics of N_2_O production in low-oxygen waters^15^. More fully parameterized versions of the ΔN_2_O/AOU that allow the yield to change as a function of oxygen do a better job but still struggle at the transition (notionally 4 μmol O_2_ l^−1^) from production to consumption of N_2_O (ref. 15). Here our mixed-effects modelling approach has enabled us to characterize a population estimate for the exponential increase in N_2_O production as a function of decreasing oxygen, from 1 μmol O_2_ l^−1^ to ∼30 μmol O_2_ l^−1^, without the confounding effects of individual site characteristics or, indeed, the need to invoke different metabolic pathways either side of an oxygen threshold.

The original version of the model by Babbin *et al.*^5^ required net production of N_2_O from both classic nitrification and denitrification to generate the typical 100 nmol l^−1^ peaks in N_2_O. Here, the orginal formulation of Babbin *et al.*, however, generated net accumulation of 20–30% N_2_O (10–60 nmol l^−1^ over 30 days) below the pycnocline, whereas with our single process variant the model only gained 1.2%. This suggests that the dynamics of N_2_O production in the two studies were fundamentally different. Indeed, we did not measure any production of ^15^N_2_ in any of the oxygen treatments, that is, none of the ^15^N_2_O from the reduction of ^15^NO_2_^−^ was further reduced to ^15^N_2_, even at 1 μmol O_2_ l^−1^, which is a key feature of the Babbin model. That, along with the non-binomial distribution of ^15^N in our N_2_O, relative to the NO_2_^−^ pool, discounts denitrification as the primary source of N_2_O here and it is redundant in our model.

The two methods that we used to estimate sea to air exchange (17 μmol N_2_O m^−2^ d^−1^, on average from the N_2_O–CO_2_ field data and a 1D directly parameterized model) agreed very well with each other and with those in the literature—despite different approaches. For example, 13 μmol N_2_O m^−2^ d^−1^ has previously been taken as representative of the ETNP^5^, while a broader range of 5–31 μmol m^−2^ d^−1^ has been estimated for the tropical south Pacific^2^,^38^. What is important for estimating the contribution from the ocean to the global N_2_O budget is the respective area of OMZ used for any extrapolation. The latter is partly defined by the concentration chosen for oxygen at which the microbiology either produces or consumes N_2_O and this is contentious^5^,^6^. Here we have measured a clear exponential increase in N_2_O production with decreasing oxygen between 1 to 30 μmol O_2_ l^−1^ and apply this to regions of the ocean defined as OMZs by oxygen minima below 20 μmol O_2_ l^−1^ (ref. 30). Applying our average rate to the OMZ of the ETNP (12.4 × 10^12^ m^2^ including a coastal strip making up only ∼3.4% of the area) and the entire global extent of OMZs (30.4 × 10^12^ m^2^) generates 2.1 Tg N y^−1^ and 5.1 Tg N y^−1^, respectively, as N_2_O. The latter of which agrees very well with estimates of approximately 5.8 Tg N y^−1^ derived from the oxygen-sensitive model of Nevison *et al.*^15^

Modelling our single response of a predominantly archaeal-driven hybrid N_2_O formation process not only accurately reproduces the distribution of N_2_O over the top 350 m of the water column but this single response can also balance our high-resolution estimates of sea to air exchange for N_2_O. Hence, a significant source of N_2_O that has for a long time now been ascribed to bacterial-mediated ammonium oxidation leaking N_2_O under oxygen stress, can better be described by an archaeal-driven hybrid N_2_O formation process exploiting the niche of low oxygen waters, at the margins of an OMZ.

## Methods

### Site-specific water column profiles and underway *p*CO_2_ data

A standard conductivity–temperature–depth rosette (24 Niskin (20 litres) and full Sea-Bird 24 electronics (salinity, density, O_2_, temperature and so on) was used to collect and characterize the water at each site between 5 and 4,000 m. The distribution of N_2_O, CO_2_ and NO_2_^−^ was measured as described previously^4^, except that the GC also had a hot-nickel catalyst and flame-ionization detector to quantify CO_2_ after rapid equilibration and reduction to CH_4_ (ref. 39). High temporal resolution measurements of *p*CO_2_ in surface seawater and atmosphere were also made every 5 min using an underway instrument (see below).

### Production of N_2_O as a function of oxygen and gene abundance

We measured the production of ^15^N-N_2_O at two depths, both within and beneath the oxycline, at each of the six sites (Supplementary Table 1). Seawater was drained from a Niskin into 4 litre Nalgene bottles and sparged for 20 min to generate six oxygen treatments (Table 1). Seawater was then dispensed under pressure into 4 × 1 litre clear glass moulded infusion vials (Laboratory Precision Limited), except for the Ambient treatments, which went directly into the 1 litre vials. Oxygen (50 μm calibrated electrode, Unisense) and temperature were measured and, following up on studies in the Arabian Sea^4^, the vials spiked with ^15^N-NO_2_^−^ ([10 μM], 98 atom%, Sigma, see Supplementary Table 1 for ^15^N atom % in each of the 12 sets of experiments). It is important to appreciate that all published work to date that uses ^15^N to trace the production of either N_2_O or N_2_ applies a ‘tracer' at concentrations in excess of apparent *K*_m_ values for these processes, that is, typically 5–10 μmol NO_2_^−^ l^−1^ spike, compared with 1–2 μmol NO_2_^−^ l^−1^ *k*_m_ and, as such, should be considered as potentials^6^,^34^,^40^. The vials were then sealed and incubated in the dark, at 12 °C, for 72 h as previously^4^. Later, bacterial activity was stopped in three of the vials by the addition of 6 ml formaldehyde (36% v/v), while the fourth vial was used to measure oxygen and the water then filtered (Supor, *ø*=47 mm, 0.2 μm pore size filters). The filters were immediately frozen in 2 ml cryovials, in liquid nitrogen, and stored at −80 °C for later extraction of nucleic acids (see below). Production of ^15^N_2_O and ^15^N_2_ was measured in the three remaining vials against reference samples for each of the treatments, or natural abundance, by mass spectrometry (see below and Nicholls *et al.*^4^). The data for each triplicate were then averaged and the mean value compared with its corresponding, single measure of functional gene abundances. Genes targeted with a potential role in N_2_O production were: β- and γ-proteobacterial *amoA*; bacterial *nirS*, bacterial *nirK*, archaeal *nirK*, archaeal *amoA* (here *AamoA*) and, in addition, general bacterial and archaeal Marine Group I 16S rRNA genes (see Supplementary Table 4 for primer sets). A combination of the large 1 litre glass vials and multiple oxygen treatments precluded a full time series incubation in each of the 12 N_2_O experiments (1,400 bottles versus 280). We did, however, measure N_2_O production at 2, 4, 9, 18, 36 and 72 h at two sites, for two oxygen treatments during a subsequent cruise to check that our single time point incubation was not overestimating production.

### Mass spectrometry for ^15^N_2_O and ^15^N_2_ and rate calculations

All the samples were transferred under constant temperature back to the home laboratory in London and were brought to 22 °C before processing. Two subsamples of the 1 litre vials were forced out under helium and transferred to either a helium-filled 12 ml gas-tight vial (Exetainer, Labco), for ^15^N_2_ analysis, or a helium-filled 20 ml gas-tight vial (Gerstel and 20 mm butyl rubber stoppers and aluminium seals, Grace—Alltech) for ^15^N_2_O analysis. The 20 ml vials ended up with 10 ml of seawater and 10 ml of helium headspace to which we added a carrier of 3 nmol N_2_O, as sparging with the compressed air, N_2_ and O_2_ treatments effectively removed all of the natural N_2_O from the samples. These were then analysed for enrichment of both single- and dual-labelled ^45^N_2_O and ^46^N_2_O, respectively, against seawater samples (collected on the cruise) sparged with the five treatment gases, or, in the case of the ambient treatment, reference samples of seawater, using a trace gas pre-concentrator unit (PreCon, Thermo-Finnigan)^4^. Calibration was performed against known amounts of N_2_O (98 p.p.m.; BOC), and it was linear (*r*^2^=0.998) over the range 0 to 20.72 nmol N_2_O absolute (∑^44^N_2_O, ^45^N_2_O and ^46^N_2_O).

After bringing the remaining 12 ml gas-tight vials to 22 °C, a helium headspace (1 ml) was added and the vials shaken by hand and left overnight on rollers (Spiramix) to allow N_2_ gas to equilibrate between the water phase and headspace. Samples of the headspace (100 μl) were then analysed for enrichment in ^15^N_2_ by injection (Multipurpose Sampler MSP2, Gerstel) into an elemental analyzer (Flash EA 1112, Thermo-Finnigan), interfaced with the continuous flow isotope ratio mass spectrometer (CF-IRMS)^4^. Calibration was performed at the beginning of each run with known amounts of oxygen free nitrogen gas (BOC) in seawater collected on the cruise, in the range of 0 to 12.6 μmol N_2_ absolute (∑^28^N_2_, ^29^N_2_ and ^30^N_2_). Values for the production of ^29^N_2_ and/or ^30^N_2_ were calculated as excess over the production in the time zero ‘reference' samples^41^.

We used ^15^NO_2_^−^ to trace the production of N_2_O as per our previous work in the Arabian Sea^4^ and present two principle methods for calculating the total production of N_2_O in response to oxygen. In the first method, given that the archaea appear to lack Nor, we assume that they cannot make N_2_O purely from exogenous NO_2_^−^ and that any measured production of *p*^46^N_2_O (that is, 2 × ^15^NO_2_^−^) must be due to canonical denitrification reducing NO_2_^−^ as far as N_2_O. Then, any production of N_2_O that we cannot account for by canonical denitrification with exogenous NO_2_^−^ we assign to hybrid N_2_O formation, as in the most recent models for Thaumarchaeotal ammonia oxidation^22^. In the second method, we assume that all of the measured production of N_2_O is due to a classic bacterial-type mode of nitrifier–denitrification, with random isotope pairing of ^14^NO and ^15^NO upstream of the production of N_2_O.

We calculate the overall production of N_2_O that we assume to be owing to canonical denitrification of exogenous NO_2_^−^ according to:


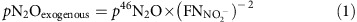


where FN_NO2−_ is the fraction of ^15^N in the NO_2_^−^ pool (Supplementary Table 1) in each set of incubations, determined by difference^34^, and we ignore any turnover by either ammonium or nitrite oxidation, which is shown to be negligible relative to the size of the NO_2_^−^ pool (See the ‘Discussion' section and Supplementary Table 2). We then used the measured amount of dual-labelled *p*^46^N_2_O to predict the expected amount of single-labelled *p*^45^N_2_O_exp_ for canonical denitrification according to^40^:


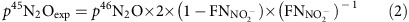


We would then argue that any production of *p*^45^N_2_O above *p*^45^N_2_O_exp_ cannot be solely due to reduction of external NO_2_^−^, and must be due to ^15^N pairing with an alternative source of ^14^N (for example, ^15^NO from ^15^NO_2_^−^, pairing with ^14^NH_2_OH in archaeal hybrid N_2_O formation^22^) which, for simplicity, we refer to as endogenous N_2_O:


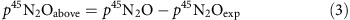



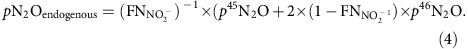


The first estimate of total production of N_2_O in our incubations with ^15^NO_2_^−^ is then the sum of the two former products:


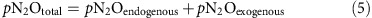


Hence, the calculation of *p*N_2_O_total_, *p*N_2_O_endogenous_ and *p*N_2_O_exogenous_ with ^15^NO_2_^−^ is synonymous to that for total N_2_, anammox and denitrification, respectively, in all other work measuring the production of N_2_; though the biological context is not^40^. The alternative formulation assumes that all of our measured production of N_2_O was dominated by a classic bacterial-type mode of nitrifier–denitrification, with random isotope pairing of ^14^NO and ^15^NO upstream of the production of N_2_O (ref. 22) and we can calculate an alternative *p*N_2_O_total_′ according to^42^:


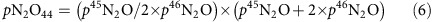



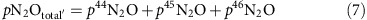


### Molecular analysis

In the home laboratory, each Supor filter was cut in half and one half was placed into a 2 ml sterile screw-cap tube, containing *ø*=0.1 mm glass beads. The following solutions were then added to each tube: 700 μl of 120 mmol l^−1^ sodium phosphate (pH 8.0) plus 1% (w/v) acid-washed polyvinylpolypyrrolidone, 500 μl of Tris-equilibrated phenol (pH 8.0), and 50 μl of 20% (w/v) sodium dodecyl sulfate. The extraction process involved bead beating and passing the samples through hydroxyapatite and Sephadex G-75 spin columns, to separate nucleic acids from proteins and salts^43^. Nucleic acids were resuspended in 50 μl of TE (10 mmol l^−1^ Tris, 1 mM EDTA [pH 8.0]) and stored at −80 °C.

The extracted DNA was used for quantification of functional genes (primer details are shown in Supplementary Table 4). Quantitative real-time PCR was performed in a Bio-Rad CFX96 Real-Time System. The reaction was performed in duplicate in a final volume of 15 μl, which contained 7.5 μl of SensiFAST SYBR No-ROX mix (2 × ) (Bioline), 200 nmol l^−1^ of each primer and 1 μl of 10 times diluted DNA. The conditions for all reactions were as follows: 95 °C for 3 min; 40 cycles of 95 °C for 0.05 min and 60 °C for 0.30 min; 95 °C for 0.05 min; 65 °C for 0.05 min and a final step of 95 °C for 0.5 min. Absolute quantification of the targeted genes was performed with a series of 10-fold standard dilutions, using the CFX Manager version 2.0 software (Bio-Rad). Standards for bacterial 16S rRNA, *nirS* and *nosZ* genes were derived from *Pseudomonas brenneri* DSM15294; environmental PCR products were used for bacterial *amoA*, *AamoA, nirK, AnirK and MG1 16S*. Samples with Cq values that were the same or greater than those of the no template controls were assumed to be below the limit of detection (LOD). In each of these cases, the calculated LOD for the particular qPCR plate was used as the value for that sample (maximum LOD=171 copies ml^−1^). Specificity of the *AnirK* PCR was assessed by sequencing product from a number of sites. All showed that the PCR assay was specific for its target gene (data not shown).

### Nitrification

To account for any possible turnover of the ^15^NO_2_^−^ pool in our 72 h ^15^N_2_O incubations, we incubated additional water under ambient oxygen (1 to 23 μmol O_2_ l^−1^) from the second depth at each site (Supplementary Table 1). Water was sampled into 1 litre vials, allowed to overflow three times, sealed, brought to 12 °C and then, without any sparging, pushed out (2 mm Teflon tubing) under helium into the bottom of 12 ml, gas-tight vials (Exetainer, Labco), overflowed three times and sealed. The vials were then enriched from concentrated stocks (Sigma, sparged with OFN) to [10 μmol l^−1^], in quadruplets, with either ^15^NO_2_^−^ or ^15^NH_4_^+^. Ammonia oxidation was estimated from the net accumulation of ^15^NO_2_^−^ after the addition of ^15^NH_4_^+^, single time point incubations (96 h); nitrite oxidation from net accumulation of ^15^NO_3_^−^ over 3, 6, 12, 24, 48 or 96 h from ^15^NO_2_^−^ and overall net nitrification from the accumulation of total ^15^NO_*x*_^−^ after 96 h from ^15^NH_4_^+^. The samples were fixed (50 μl 50% (w/v) ZnCl_2_) and production of ^15^NO_*x*_^−^, ^15^NO_2_^−^ or ^15^NO_*x*_^−^ measured with a sulphamic acid assay at the University of Southern Denmark.

### Modelling N_2_O production

We formulated a simple 1D model of N_2_O (1 m depth resolution), coded in R. The model is largely based on the parameterizations given by Babbin *et al.*^5^ encompassing physical processes (upwelling, vertical diffusion and implicit gas exchange of N_2_O) as well as biological production of N_2_O. Vertical transport was parameterized according to Fickian diffusion with a diffusivity *K*_z_ of 4 × 10^−4^ m^2^ s^−1^ at the surface, decreasing linearly to 4 × 10^−5^ m^2^ s^−1^ at 10 m and remained constant thereafter apart from the pycnocline (20–48 m depth) where *K*_z_ was 1 × 10^−5^ m^2^ s^−1^. This *K*_z_ profile effectively simulated near surface turbulence while the remaining water column was dominated by diffusive processes. An upwelling velocity (*w*_up_) of 8 × 10^−7^ m s^−1^ and a particle sinking velocity (of 1.2 × 10^−4^ m s^−1^ (ref. 5) were used.

The model resolved the upper 400 m of the water column at 1 m depth resolution and 1 min time intervals. Boundary conditions at the surface and at 400 m were fixed and prescribed by the respective averages from our profiles. This average profile also described initial conditions for NO_3_^−^, PO_4_^3−^, N_2_O and O_2_. Particulate Organic Carbon (POC) values for the ETNP were taken from the literature^44^,^45^, with a surface concentration of 3 μmol l^−1^, a sub-surface maximum of 5 μmol l^−1^ at 32 m and decreasing thereafter to 1.3 μmol l^−1^ at 400 m. Model POC remineralization (POC_rem_) was parameterized as a first-order process with a rate constant of 5 × 10^−7^ s^−1^. POC production at the surface was implicit via the fixed boundary concentration as in Babbin *et al.*^5^ In addition, we parameterized POC production (_p_POC_Z_) at depth (*Z*) as a function of the upwelling NO_3_^−^ flux and light attenuation:


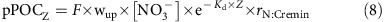


where *F* is the ratio of upwelled NO_3_^−^ used by primary producers (0.2), [NO_3_^−^] is the concentration of NO_3_^−^ at depth *Z*, *K*_d_ is the light attenuation coefficient (0.09 m^−1^) and *r*_N:Cremin_ is the N:C ratio production/remineralization (*r*_N:Cremin_=16/106). The value of *K*_d_ was chosen as it gave a subsurface _p_POC_Z_ maximum which was consistent with the positions of the subsurface POC- and chlorophyll-concentration maxima at the base of the mixed layer as observed during our cruise. NO_3_^−^, PO_4_^3−^ and O_2_ were linked to POC production/remineralization according to Redfield stoichiometry, as in Babbin *et al.*^5^ O_2_ consumption followed a respiratory ratio of ∼1.4 (*r*_O:Crem_=150:106).

Production to consumption of N_2_O was parameterized for two separate variants of the model: (i) according to Babbin *et al.*^5^ and (ii) as a function of O_2_ concentration as described here. All processes except those producing N_2_O were identical in both the models. In our second variant, we parameterized model N_2_O production (*p*N_2_O in nmol m^−3^ d^−1^) using the estimates for *a* and *b* from our nonlinear mixed-effects models (M2 and M5, Table 2 and equation 12):


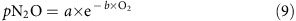


Note that the original Babbin formulation included an [O_2_]-dependent Heaviside function which terminated N_2_O production when [O_2_]<0.4 *μ*mol L^−1^. Here, as oxygen was always above 0.4 *μ*mol O_2_ L^−1^ it was redundant and not included in our variant of the model.

### Estimating N_2_O exchange using high-resolution *p*CO_2_ data

High temporal resolution measurements of *p*CO_2_ in surface seawater and atmosphere were made every 5 min using an underway instrument (PML Dartcom Live *p*CO_2_. UK.^46^,^47^) with the ‘vented' equilibrator modification^46^. The equilibrator was fitted with two platinum resistance thermometers (Pico Technology, model PT100) and a water-jacket supplied with seawater from the ship's underway seawater system. A seawater flow of 1.6 litres min^−1^ was maintained through the main equilibrator. The average warming between the ship's underway seawater intake and the equilibrator was 0.2±0.1 °C. Atmospheric measurements of CO_2_ were taken from an intake located on the foremast. Both gas streams from the equilibrator headspace and the air inlet were dried in a Peltier cooler (−20 °C). Mixing ratios of CO_2_ and water in the marine air and equilibrator headspace were determined by non-dispersive infrared dection (LI-840, LI-COR). Measurements were referenced against secondary calibration gases (BOC Gases, UK) with known CO_2_ mixing ratios (257.6, 373.4 and 463.5 μmol CO_2_ per mole) in synthetic air mixtures (21% oxygen and 79% nitrogen). All calibration gases were calibrated against certified primary standards from the National Oceanic and Atmospheric Administration (244.9 and 444.4 μmol CO_2_ per mole). The *p*CO_2_ system described here showed high consistency with a similar *p*CO_2_ system and *p*CO_2_ calculated from independent TA, DIC and pH during ‘at sea' inter-comparison^48^. Sampling was carried out continuously (every 5 min), with the exception of periods for maintenance. See Supplementary Fig. 7 for a summary of the *p*CO_2_ and wind data and resultant efflux estimates.

Then, for the samples for which high-resolution seawater *p*CO_2_ (*p*CO_2_sw) data were available but in which N_2_O was not directly quantified, we predicted molar seawater N_2_O concentrations (N_2_Osw) using the linear relationship between N_2_Osw and the molar seawater concentration of CO_2_ (CO_2_sw; Supplementary Fig. 1). To do this, we first estimated the CO_2_ and N_2_O solubility for each sample (mol kg^−1^ atm^−1^; refs 49, 50). CO_2_sw for each sample was next calculated as the product of its *p*CO_2_sw and corresponding molar solubility. Each resulting molar N_2_Osw concentration was then converted to *p*N_2_Osw by dividing it by the calculated, corresponding N_2_O molar solubility. Atmospheric *p*N_2_Oatm was taken as the average for samples collected from the bow of the ship throughout the ∼6 week cruise (348±6 natm s.e., *n*=35) and the corresponding N_2_O flux estimated from the high-resolution CO_2_ fluxes calculated using the average 12 h wind speed:


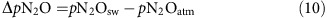



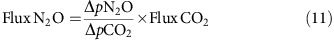


### Statistical analyses

All the analyses were conducted in *R* (ref. 32) following procedures largely described in ref. 51. We began with linear mixed-effects models treating oxygen as a categorical variable and modelling N_2_O production as an additive, linear function of the six oxygen treatments (Table 1). With the linear mixed-effects models, we fitted the oxygen treatment as a fixed effect and included random intercepts for each of the 12 experiments, comparing models with and without ‘oxygen' with likelihood ratio testing. Given that there was clear spread within the oxygen data, we then used nonlinear mixed-effects models to model ^15^N_2_O production as a continual, exponential function of oxygen:


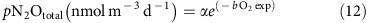


Where *p*N_2_O_total_ comes from equations (1, 2, 3, 4, 5) and O_2_exp is the measured concentration of oxygen (μmol l^−1^) in each incubation bottle and total production of N_2_O is that measured in each bottle at the end of its incubation. For the 12 sets of experiments analysed using nonlinear mixed-effects models, we either fitted both the intercept (*a*, that is, maximum N_2_O production) and sensitivity (*b*, that is, response to oxygen) as random effects, or, *a* and *b*, each individually, and compared model fit in each case with the Akaike Information Criterion (AIC). Relationships between these ‘random' elements, that is, variance not explained by experimental oxygen and other possible explanatory variables (for example, gene abundance) were explored visually (at the 12-experiment, group level, *n*=12) and then more rigorously using multiple regression and the entire, linearized and centred (*x*_c_) data set (natural log, *x*_c_=*x*−*x*_mean_, *n*=70). Here, we judge the simplest model (that is, just oxygen) against more complex models (oxygen plus single or multiple functional gene abundance) also using likelihood ratio testing.

### Data availability

The data that support the findings of this study are available from the authors on reasonable request, see author contributions for specific data sets.

## Additional information

**How to cite this article:** Trimmer, M. *et al.* Nitrous oxide as a function of oxygen and archaeal gene abundance in the North Pacific. *Nat. Commun.* **7,** 13451 doi: 10.1038/ncomms13451 (2016).

**Publisher's note**: Springer Nature remains neutral with regard to jurisdictional claims in published maps and institutional affiliations.

## Supplementary Material

Supplementary Information Supplementary Figures 1-8, Supplementary Tables 1-4 and Supplementary References

Peer Review File

## Figures and Tables

**Table 1:** Measuring the production of N_2_O as a function of oxygen.

**Treatment**	**p.p.m.**	**Balance**	**Final in water (μM)**		
			**O**_**2**_min*	**O**_**2**_max*	**N**_**2**_**O**†
**1** Air-100% saturated	NA	NA	293	322	∼0.01
**2** Ambient	NA	NA	1.0	199	∼0.01–0.1
**3** N_2_ (OFN)	999,000	NA	2.2	6.0	0.00
**4** N_2_O	2	N_2_	1.8	5.6	∼0.05
**5** O_2_	7,500	N_2_	12.9	23.2	0.00
**6** N_2_O+O_2_	2 and 7,500	N_2_	12.9	17.3	∼0.05

NA, not applicable.

^*^Minimum and maximum oxygen concentrations measured for each treatment from the 12 sets of replicate experiments.

^†^Estimated N_2_O concentration in each treatment.

The experiments were performed with water from two depths (Supplementary Table 1) at six sites (*n*=12 experiments) and with six treatments (1–6). For treatment 1, the samples were sparged with compressed air and for treatment 3, with oxygen free nitrogen (OFN, 99.9%). Samples for treatments 4, 5 and 6 were sparged with each special gas as indicated. Ambient treatment 2 was simply unadulterated seawater drained straight from a Niskin into 1 litre vials. As we did not perform treatment 1 at site 1, we have a total of 70 independent measurements of the production of N_2_O as a function of oxygen: 1 site × 2 Depths × 5 treatments+5 sites × 2 Depths × 6 treatments, *n*=70.

**Table 2:** Output from the nonlinear mixed-effects modelling of N_2_O production as an exponential function of experimentally induced decreasing oxygen.

**Model**	**Parameter**	**Estimate**	**s.e.**	***t*****-value**	***P*** **value**	**Random effect**	**Variance structure**	**AIC**
M1	*a*	123.94	24.61	5.11	<0.001	Yes	No	746
	*b*	0.0535	0.010	5.12	<0.001	No		
M2	*a*	120.57	24.17	4.99	<0.001	Yes	Yes	730
	*b*	0.0514	0.011	4.37	<0.001	No		
M3	*a*	133.44	22.95	5.81	<0.001	Yes	Yes	728*
	*b*	0.0732	0.015	4.72	<0.001	Yes		
M4	*a*	137.44	23.09	5.95	<0.001	Yes	No	742
	*b*	0.0780	0.015	5.05	<0.001	Yes		
M5	*a*	612.62	149.7	4.09	<0.001	Yes	Yes	NA
	*b*	0.0681	0.009	6.84	<0.001	No		

NA, not applicable.

^*^M3 had the lowest AIC score but its random intercept (*a*) and exponent (*b*) were highly correlated (*r*=−0.99), which suggested that the model was over parameterized and therefore M2 was taken as the most parsimonious fit to the data.

The goodness of fit for each model to the data was judged using the Akaike Information Criterion (AIC), where a lower value indicates a better fit. M2 and M3 were improved further by the addition of a power variance structure at the level of each of the 12 experiments. Note that M5 was fitted to data calculated assuming a bacterial mode of N_2_O production with random isotope pairing of ^14^NO and ^15^NO using equations (6 and 7) (*p*N_2_O_total_′) and, as such, comparison with the other models using AIC is not appropriate. In addition, note the far higher intercept in M5, which manifests as over production of N_2_O in Fig. 5b.
