# Supplementary material for: Nitrous oxide as a function of oxygen and archaeal gene abundance in the North Pacific
Source: Nat Commun. 2016 Dec 1;7:13451. doi: 10.1038/ncomms13451 (PMC5146275; doi:10.1038/ncomms13451)
Supplement: Supplementary Information — Supplementary Figures 1-8, Supplementary Tables 1-4 and Supplementary References [file ncomms13451-s1.pdf]

Supplementary Figure 1. Additional water column chemistries. **a,** water column profiles for N_2O (green), O_2 (red) and NO_2^- (blue) to 700m for all six sites combined. **b,** carbon dioxide, as for **a,** and **c,** correlation between N_2O and CO_2 over the top 45m (with a 1st order linear regression and 95% confidence intervals marked in blue). We used the latter relationship to extrapolate our site specific, profile measures of N_2O to the greater temporal and spatial resolution captured by the high-resolution (n = 4820) underway pCO_2 measurements (*see* Supp. Fig. 6 and Methods).

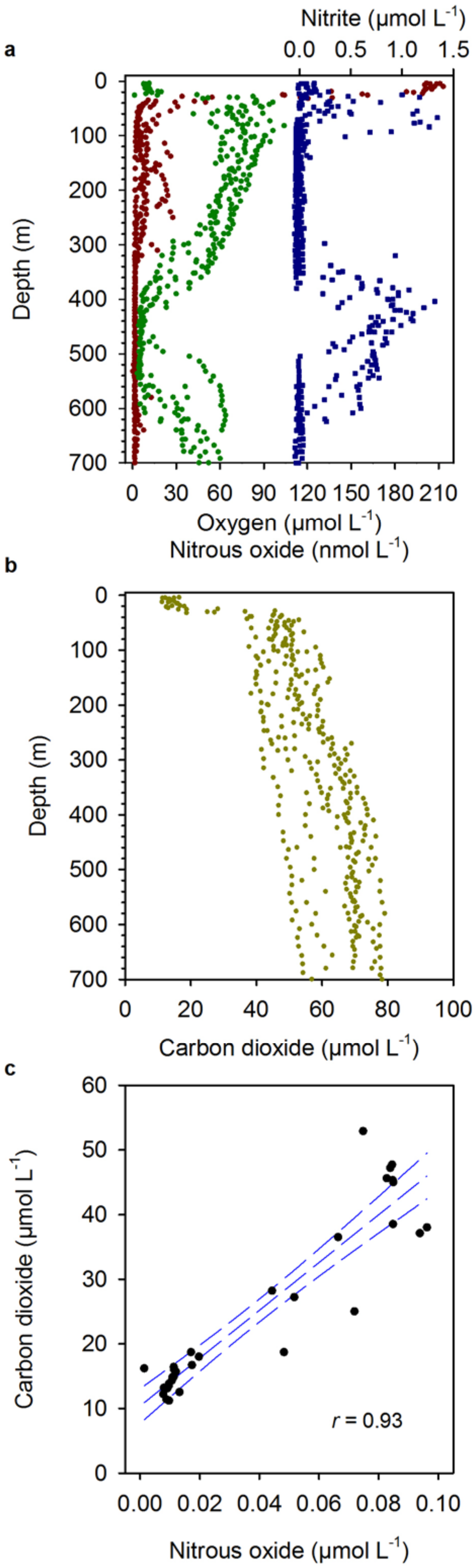

Supplementary Figure 2. Our measured profiles of N_2O (nM) in the top 500m (open red circles) against 13 data sets (open black circles) in the MEMENTO database[1] from the tropical North Pacific. **a.** from, bottom left, approximately 0°N to, top-right, 28°N and **b.** from, bottom-left, 167°W to, top-right, 80°W (in negative easterlies). Note the distinct drop in N_2O in the top 100m beyond approximately 155°W to 165°W (bottom two, left-panels) towards the margins of OMZ[2] and that data from our transect down 92.5°W appears twice because of other transects in the database bisecting it from NE to SW.

a.

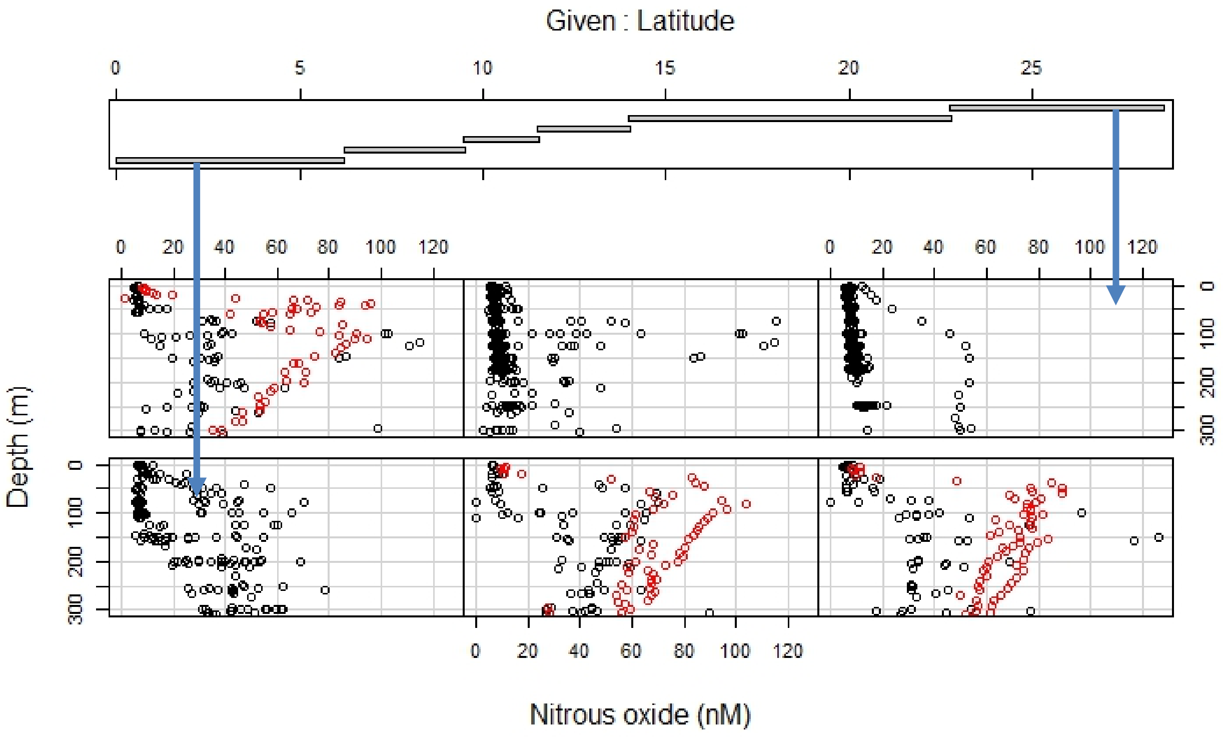

b.

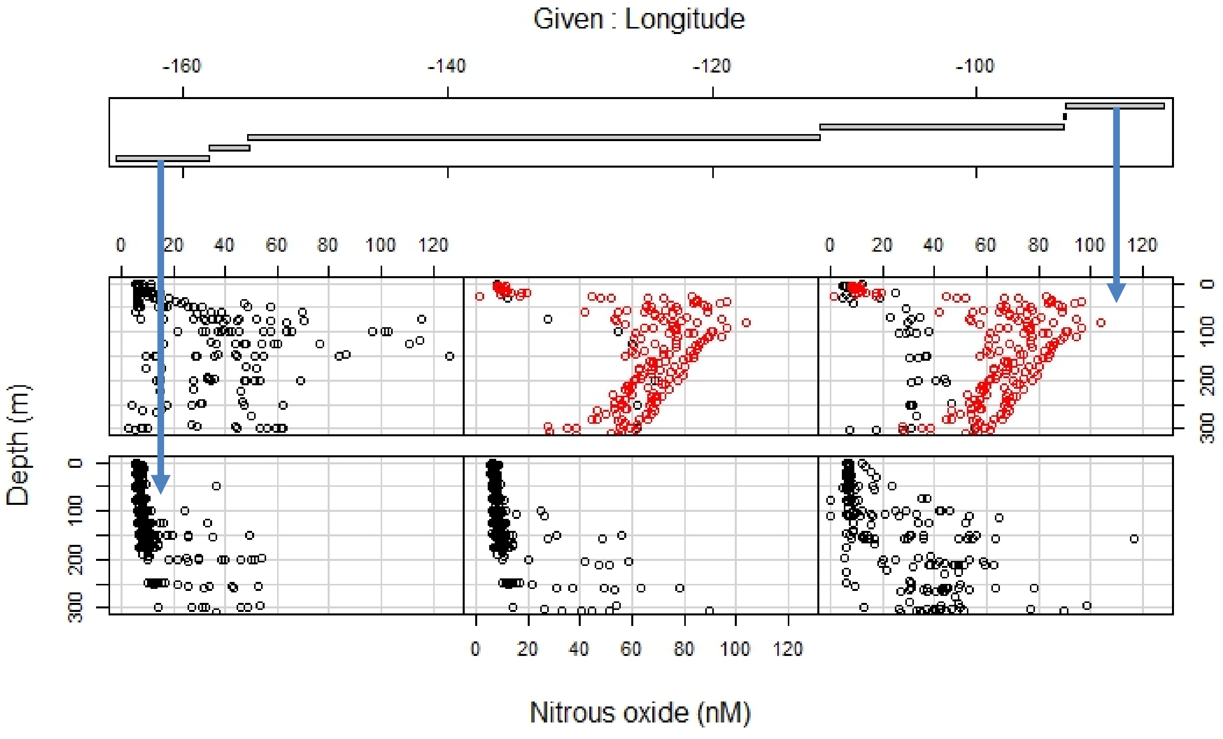

Supplementary Figure 3. The complete output from the non-linear mixed effects model (M2) of total N_2O production as a function of oxygen in each of the 12 experiments (6 Sites x 2 depths (m)). The model allows the magnitude of N_2O production to vary randomly between the 12 groups of experiments (i.e. it treated each group level intercept [*a*] as a random effect, pink lines), while keeping the response to oxygen [*b*] constant, in order to derive an overall population estimate (fixed effect, blue line) for the production of N_2O as a function of oxygen. We then used these final parameter estimates ([a] and [b]) as inputs to a simple 1D model to see if our argued, single process could reproduce our measured water column profiles of N_2O.

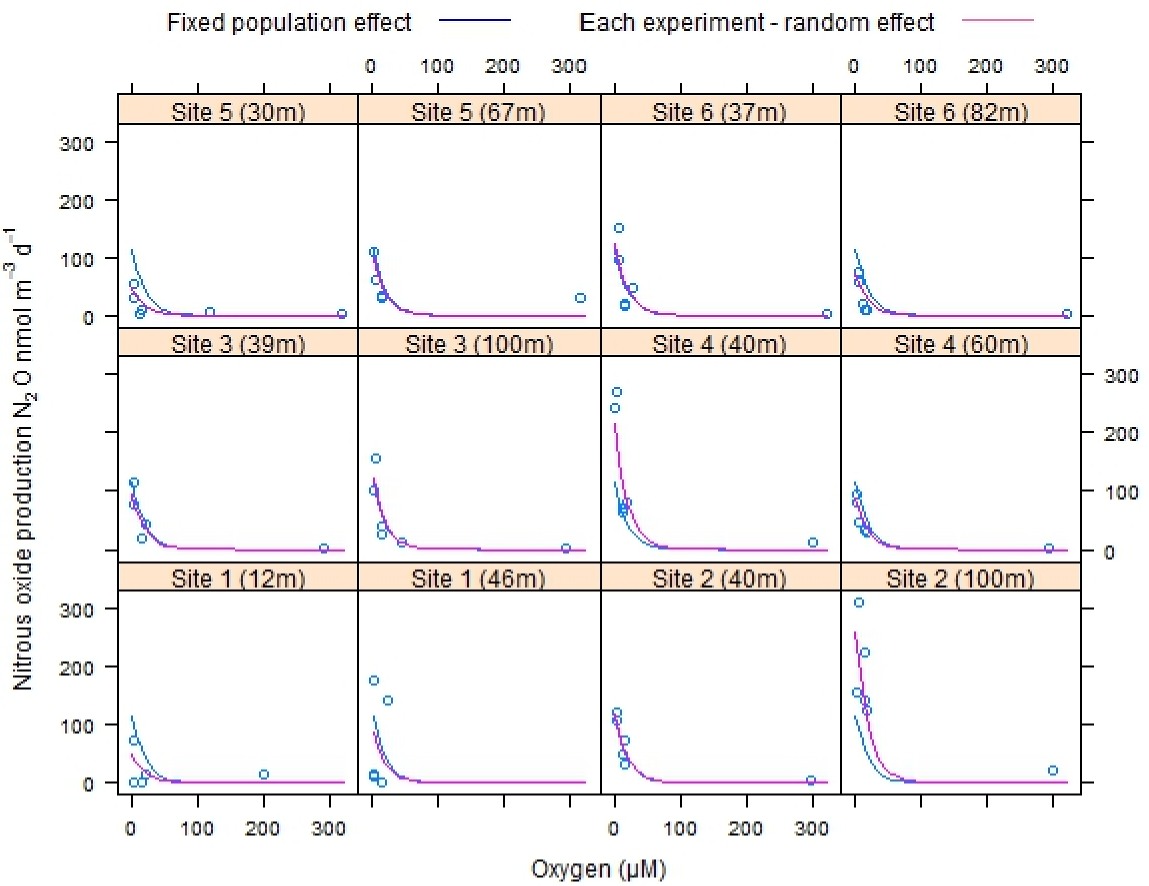

Supplementary Figure 4. A combination of the large 1L glass-vials and multiple oxygen treatments precluded a full time series incubation in each of the 12 N_2O by oxygen treatment experiments (280 versus 1400). We did, however, measure its production at 2, 4, 9, 18, 36 and 72h at two oxygen saturations and for two depths on a follow-up cruise. Where production was strongest and most linear (90m) the rates recorded ~30 nmol m^{-3} d^{-1} and 56 nmol m^{-3} d^{-1} are representative of our overall median rate of production below 30 µmol O_2 L^{-1} of 58nmol m^{-3} d^{-1}. At 60 m production was weak and markedly non-linear over 72h but not representative of our main data set at ~8nmol m^{-3} d^{-1}. In the 12 experiments making up the main study, where we only measured production after 72h, it would most likely be underestimated.

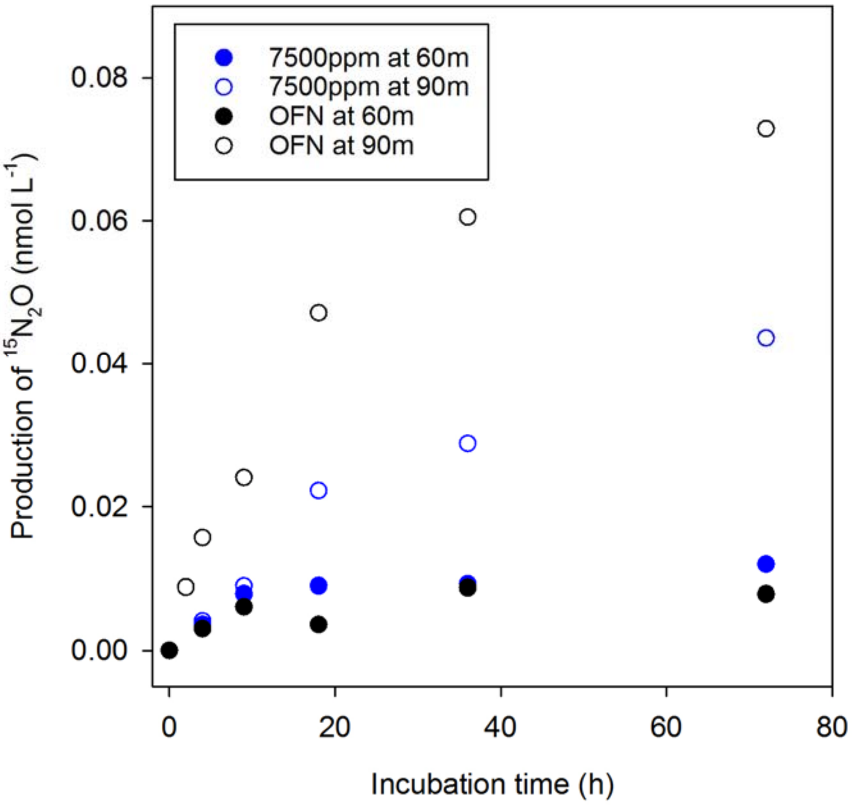

Supplementary Figure 5. Here we began by modelling total N_2O production as an exponential function of decreasing oxygen in each of the 12 experiments using a non-linear mixed-effects model. Our final selected model (M2 and Supp. Fig. 3) generates 12 random intercepts (parameter *a*, or the maximum height of pink lines in Supp. Fig. 3) which can then be explored in relation to other explanatory variables. **a.** Box-plots of the deviation in the random intercept (parameter *a*) for samples collected from above and below 30 µM oxygen i.e. less N_2O is produced on average with water from above 30 µM oxygen. Each box shows the 25th and 75th percentile, overall spread in the data and median value (horizontal line). **b.** deviation in the random intercept as a function of median gene abundance for bacterial *nirS* and *nirK* in each of the 12 experiments and **c.** as for **b.** but for archaeal *AnirK* and *AamoA*. The potential relationships in **c.** is then explored further in the main text using multiple linear regression with the entire dataset (*n*=70), not just the median values shown here.

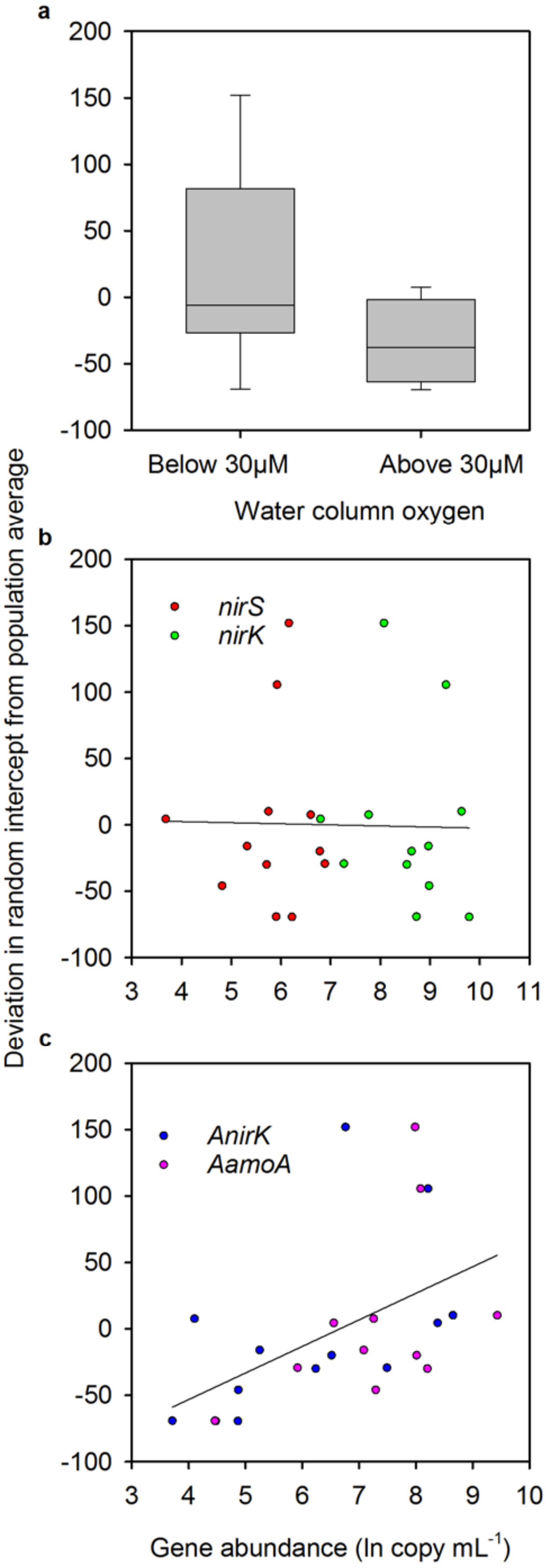

Supplementary Figure 6. Average abundances (± standard errors) for the functional genes targeted in this study along with 16S for bacteria and marine-group 1 archaea.

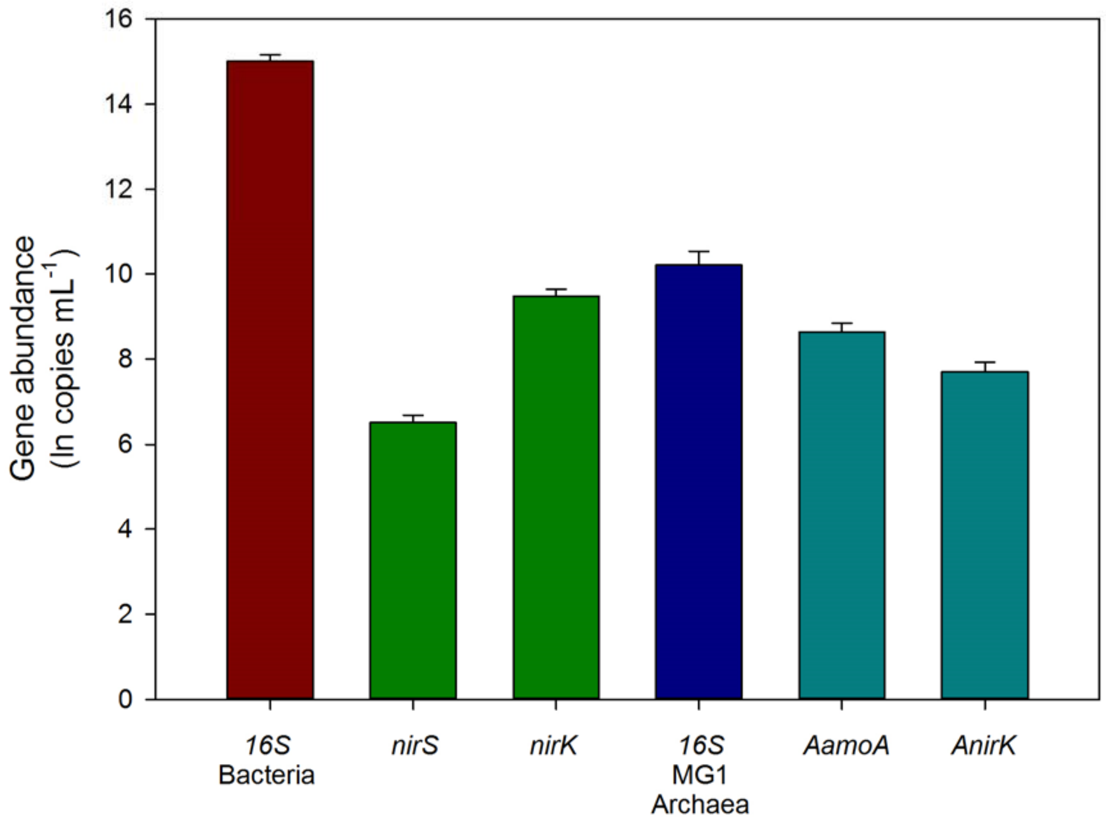

Supplementary Figure 7. Basic ordination of a principle component analysis of the four functional genes used in the modelling of N_2O production. Where copy number mL^{-1} has been log transformed (natural, ln) in each case and lnAOA stands for ln *AamoA*; other genes are as in the main text. Each pair of either bacterial (*nirS* and *nirK*) or archaeal (*AnirK* and *AamoA*) genes were positively correlated with each other but were ordinated apart in the samples. PC1 and PC2 explained 73% of the cumulative variance.

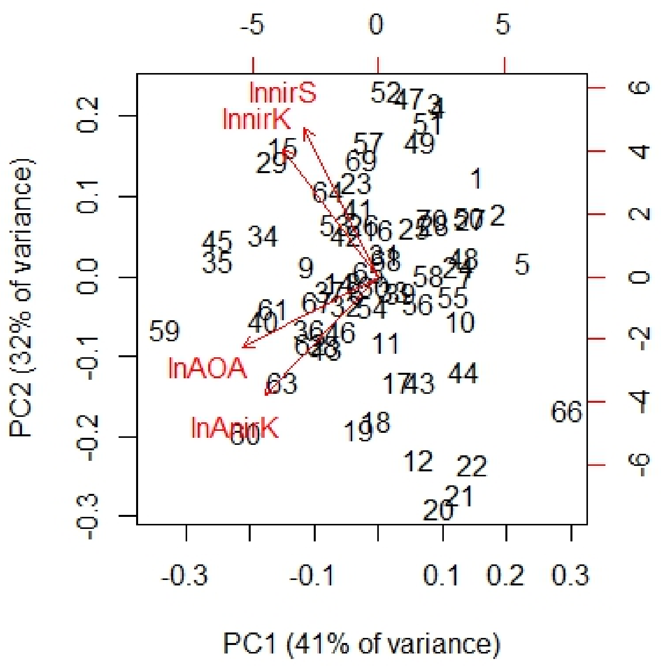

Supplementary Figure 8. Summary of underway pCO_2 measurements made on board the *RRS Discovery* along our transect from 13°N to 8°N, down 92.5°W. **a.** all 4820 seawater pCO_2 measurements (mean in blue) relative to the atmosphere (mean in black). **b.** 12h-average wind speed data, with mean (black) and 1st and the 3rd quartiles (light blue) and **c.** the corresponding estimated flux of CO_2 from **a.** & **b** with the same distributions as indicated in **b**. Average exchange for CO_2 was 7.2mmol m^{-2} d^{-1}, with a maximum of 49 mmol m^{-2} d^{-1}. These high-resolution flux estimates for CO_2 were used in combination with the correlation between CO_2 and N_2O measured in the surface waters to extrapolate a flux estimate for N_2O (Supp. Fig. 1c and Methods).

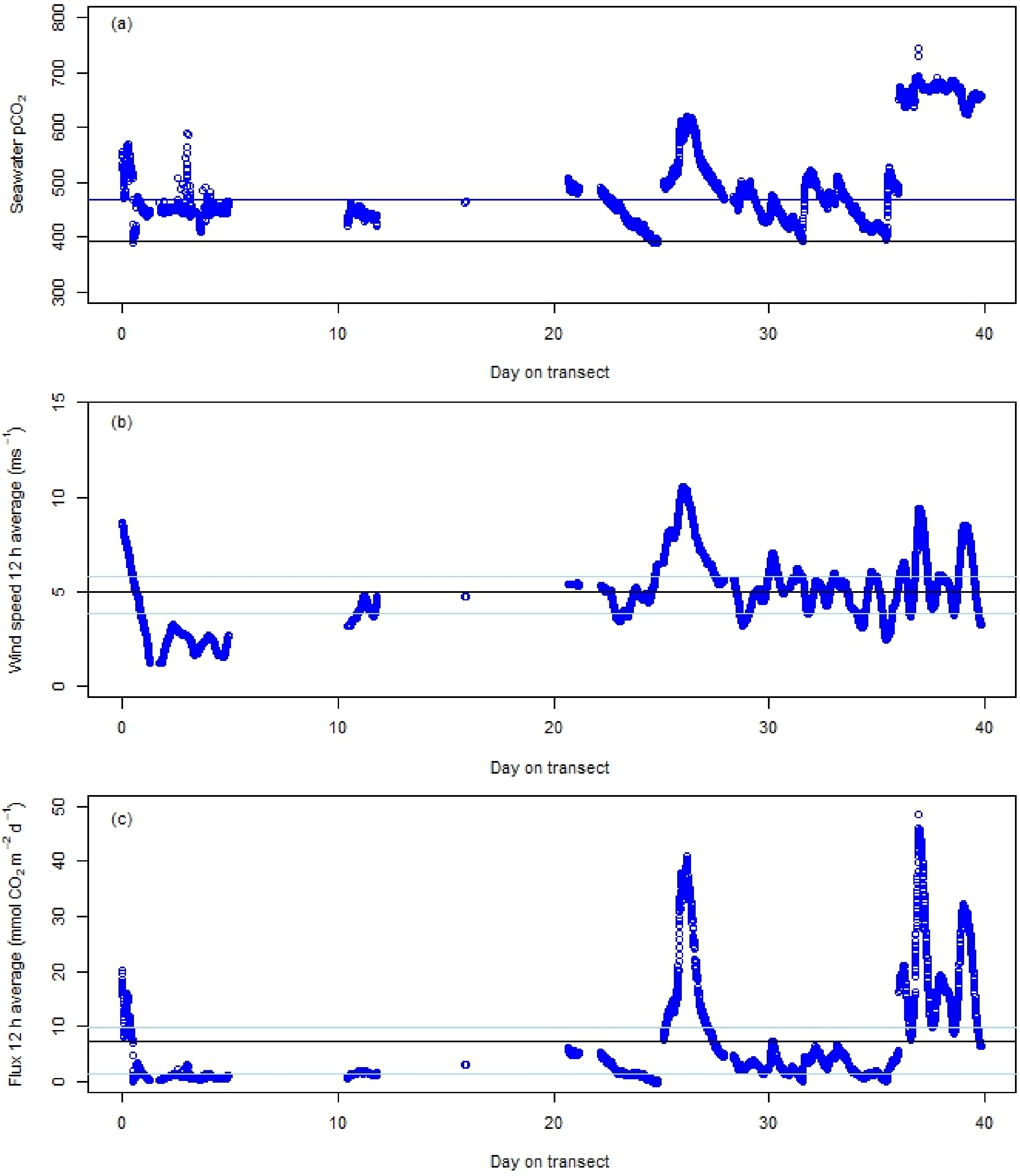

Supplementary Table 1. Principal site locations, sampling depths for the N_2O as a function of oxygen experiments and ambient oxygen and nitrite for each depth. FN_{NO2-} is the frequency of $^{15}NO_2^-$ at the start of the incubations, after the addition of 10µmol $^{15}NO_2^-$ L^{-1}. The predicted $^{45}N_2O$ is the fraction (percent) of total ^{15}N labelled gases produced ($^{45}N_2O$ and $^{46}N_2O$) predicted to be $^{45}N_2O$, assuming denitrification as the dominant source of N_2O (as per our previous work [3]), a random, binomial distribution based on FN_{NO2-} and the measured production of $^{46}N_2O$ in each experimental vial [4]. Note, after being measured for $^{15}N_2O$ each vial was also measured for any $^{15}N_2$ production and, at the deeper depth at each site, at ambient oxygen, we also estimated ammonium oxidation, nitrite oxidation and net nitrification in parallel 12 mL gas-tight vials (Methods and Supp. Table 2).

Site	Latitude (N)	Longitude (W)	Depth (m)	Ambient oxygen (µmol L^{-1})	Ambient nitrite (µmol L^{-1})	FN_{NO2-}	Predicted $^{45}N_2O$ (%)
1	12	92.5	12	199	0.08	0.972	5.37
			46	1.0	0.30	0.951	9.25
2	13	92.5	40	1.1	0.01	0.979	4.11
			100	3.7	0.03	0.977	4.47
3	11	92.5	39	10.8	0.30	0.951	9.26
			100	30.0	0.01	0.979	4.11
4	10	92.5	40	11.5	0.50	0.933	12.50
			60	4.6	0.20	0.961	7.55
5	9	92.5	30	57.0	0.20	0.961	7.55
			67	3.3	1.30	0.867	23.44
6	8	92.5	37	18.0	0.30	0.951	9.26
			82	8.0	0.10	0.970	5.77

Supplementary Table 2. Summary of rate estimates for nitrification at ambient oxygen for the deepest depth at each site in 12mL gas-tight vials. Ammonia oxidation estimated from the net accumulation of $^{15}NO_2^-$ after addition of $^{15}NH_4^+$; nitrite oxidation from net accumulation of $^{15}NO_3^-$ from $^{15}NO_2^-$ and overall net nitrification from the accumulation of total $^{15}NO_x^-$ ($^{15}NO_3^-$ combined with $^{15}NO_2^-$) also with $^{15}NH_4^+$. Note, ammonia oxidation and net nitrification were single time point incubations (96h), whereas nitrite oxidation was estimated by linear regression through multiple time point data (3, 6, 12, 24, 48 or 96 h). These data served only to estimate the turnover of the $^{15}NO_2^-$ pool (~ 10 µM) in the main N_2O by oxygen treatment experiment. ^{15}N analysis provided by the University of Southern Denmark.

Site	Depth (m)	Oxygen (µmol L^{-1})	Ammonia oxidation	Nitrite oxidation (nmol N L^{-1} d^{-1})	Net nitrification
1	46	1	1.1 ± 0.10	16.9 ± 2.6	2.6 ± 0.8
2	100	12.6	0.1 ± 0.03	14.6 ± 2.2	5.4 ± 1.4
3	100	22.6	1.9 ± 0.20	20.6 ± 2.6	2.4 ± 0.7
4	60	6.6	5.9 ± 0.20	16.1 ± 6.8	8.2 ± 1.4
5	67	5.2	1.8 ± 0.30	9.9 ± 3.9	5.4 ± 1.9
6	82	12.7	3.5 ± 0.30	36.5 ± 10.5	6.6 ± 3.1
mean			2.4 ± 0.8	19.1 ± 3.8	5.1 ± 0.9

Supplementary Table 3. Output from the multiple linear regression (mixed effects) modelling of total N_2O production as a function of oxygen and the abundance of candidate functional genes for production of N_2O. Data were first linearized by log-transformation (natural, ln) and centered ($x - x_{mean}$) and then modelled with a random intercept for each of the 12 experiments and either oxygen (O_2) and/or each functional gene as fixed effects. The goodness of fit for each model to the data was judged using the Akaike Information Criterion (AIC), where a lower value indicates a better fit. The significance of each Chi-Square value, for single or multiple combinations of fixed effects against a simpler model, was assessed using the log-likelihood ratio test.

**Model**	**Parameters included**						**AIC**	***P*(>Chisq)***
M6 (null)	1						243.9	na
M7		O_2					200.7	<0.000
M8		O_2	*nirS*				201.7	0.3294
M9		O_2		*nirK*			200.9	0.1887
M10		O_2	*nirS*	*nirK*			199.9	0.0939
M11		O_2	*nirS*	*nirK*	*AamoA*		197.0	0.0223
M12		O_2			*AamoA*		196.7	0.0142
M13		O_2	*nirS*	*nirK*	*AamoA*	*AnirK*	196.4	0.0158
M14		O_2			*AamoA*	*AnirK*	196.3	0.0152
M15		O_2				*AnirK*	196.7	0.0142

*Note, the null model M6 only has an intercept (1) for comparison with the other more complex models. M7 is relative to M6 i.e. inclusion of oxygen in the model, whereas all subsequent models (M8 to M15) are relative to M7 i.e. inclusion of functional genes in addition to oxygen in a model.

Supplementary Table 4. Primers used for quantification of genes in real-time PCRs.

**Target gene**	**Primer Name**	**Primer Sequence (5′ → 3′)**	**Reference**
16S (Bacteria)	Bact_341F<br>Bact_805R	CCT ACG GGN GGC WGC AG<br>GAC TAC HVG GGT ATC TAA TCC	Herlemann *et al.* (2011)[5]
16S (Archaea Marine Group I)	GI_751F<br>GI_956R	GTCTACCAGAACAYGTTC<br>HGGCGTTGACTCCAATTG	Mincer *et al*. (2007)[6]
nirS	cd3aF<br>R3cd	GTS AAC GTS AAG GAY ACS GG<br>GAS TTC GGY TGS GTC TTG A	Michotey *et al.* (2000)[7]<br>Throback et al. (2004)[8]
nirK(Bacteria)	F1aCu<br>R3Cu	ATC ATG GTS CTG CCG CG<br>GCC TCG ATC AGY TTG TGG TT	Hallin & Lindgren (1999)[9]
nirK (Archaea)	AnirKa-58F<br>AnirKa- 579R	ACB YTA TTC GGA AGY ACA TAC ACA<br>GYM ATT CCG TAC ATK CCG GA	Lund *et al.* (2012)[10]
amoA (*β*-proteobacteria)	amoA-1F<br>amoA-2R	GGG GTT TCT ACT GGT GGT<br>CCC CTC KGS AAA GCC TTC TTC	Rotthauwe *et al.* (1997)[11]
amoA (*γ*-proteobacteria)	amoA-3F<br>amoB-4R	GGT GAG TGG GYT AAC MG<br>GCT AGC CAC TTT CTG G	Purkhold *et al.* (2000)[12]
amoA (Archaea)	CrenamoA-23F<br>CrenamoA-616R	ATG GTC TGG CTW AGA CG<br>GCC ATC CAT CTG TAT GTC CA	Tourna *et al.* (2008)[13]

Supplementary references

1. Bange, H. W. *et al.* MEMENTO: a proposal to develop a database of marine nitrous oxide and methane measurements. *Environmental Chemistry* **6**, 195-197, doi:10.1071/en09033 (2009).
2. Paulmier, A. & Ruiz-Pino, D. Oxygen minimum zones (OMZs) in the modern ocean. *Progress in Oceanography* **80**, 113-128, doi:10.1016/j.pocean.2008.08.001 (2009).
3. Nicholls, J. C., Davies, C. A. & Trimmer, M. High-resolution profiles and nitrogen isotope tracing pairs reveal a dominant source of nitrous oxide and multiple pathways of nitrogen gas formation in the central Arabian Sea. *Limnology and oceanography* **52**, 156 - 168 (2007).
4. Thamdrup, B. & Dalsgaard, T. Production of N_2 through anaerobic ammonium oxidation coupled to nitrate reduction in marine sediments. *Applied and environmental microbiology* **68**, 1312 - 1318, doi:10.1128/AEM.68.3.1312-1318.2002 (2002).
5. Herlemann, D. P. R. *et al.* Transitions in bacterial communities along the 2000 km salinity gradient of the Baltic Sea. *Isme Journal* **5**, 1571-1579, doi:10.1038/ismej.2011.41 (2011).
6. Mincer, T. J. *et al.* Quantitative distribution of presumptive archaeal and bacterial nitrifiers in Monterey Bay and the North Pacific Subtropical Gyre. *Environmental Microbiology* **9**, 1162-1175, doi:10.1111/j.1462-2920.2007.01239.x (2007).
7. Michotey, V., Mejean, V. & Bonin, P. Comparison of methods for quantification of cytochrome cd(1)-denitrifying bacteria in environmental marine samples. *Applied and Environmental Microbiology* **66**, 1564-1571, doi:10.1128/aem.66.4.1564-1571.2000 (2000).
8. Throback, I. N., Enwall, K., Jarvis, A. & Hallin, S. Reassessing PCR primers targeting nirS, nirK and nosZ genes for community surveys of denitrifying bacteria with DGGE. *Fems Microbiology Ecology* **49**, 401-417, doi:10.1016/j.femsec.2004.04.011 (2004).
9. Hallin, S. & Lindgren, P. E. PCR detection of genes encoding nitrile reductase in denitrifying bacteria. *Applied and Environmental Microbiology* **65**, 1652-1657 (1999).
10. Lund, M. B., Smith, J. M. & Francis, C. A. Diversity, abundance and expression of nitrite reductase (nirK)-like genes in marine thaumarchaea. *Isme Journal* **6**, 1966-1977, doi:10.1038/ismej.2012.40 (2012).
11. Rotthauwe, J. H., Witzel, K. P. & Liesack, W. The ammonia monooxygenase structural gene amoA as a functional marker: Molecular fine-scale analysis of natural ammonia-oxidizing populations. *Applied and Environmental Microbiology* **63**, 4704-4712 (1997).
12. Purkhold, U. *et al.* Phylogeny of all recognized species of ammonia oxidizers based on comparative 16S rRNA and amoA sequence analysis: Implications for molecular diversity surveys. *Applied and Environmental Microbiology* **66**, 5368-5382, doi:10.1128/aem.66.12.5368-5382.2000 (2000).
13. Tourna, M., Freitag, T. E., Nicol, G. W. & Prosser, J. I. Growth, activity and temperature responses of ammonia-oxidizing archaea and bacteria in soil microcosms. *Environmental Microbiology* **10**, 1357-1364, doi:10.1111/j.1462-2920.2007.01563.x (2008).
